# The Coding and Noncoding Architecture of the *Caulobacter crescentus* Genome

**DOI:** 10.1371/journal.pgen.1004463

**Published:** 2014-07-31

**Authors:** Jared M. Schrader, Bo Zhou, Gene-Wei Li, Keren Lasker, W. Seth Childers, Brandon Williams, Tao Long, Sean Crosson, Harley H. McAdams, Jonathan S. Weissman, Lucy Shapiro

**Affiliations:** 1 Department of Developmental Biology, Stanford University, Stanford, California, United States of America; 2 Department of Cellular and Molecular Pharmacology, California Institute of Quantitative Biology, Center for RNA Systems Biology, Howard Hughes Medical Institute, University of California, San Francisco, San Francisco, California, United States of America; 3 Department of Biochemistry and Molecular Biology, University of Chicago, Chicago, Illinois, United States of America; University of California, Santa Barbara, United States of America

## Abstract

*Caulobacter crescentus* undergoes an asymmetric cell division controlled by a genetic circuit that cycles in space and time. We provide a universal strategy for defining the coding potential of bacterial genomes by applying ribosome profiling, RNA-seq, global 5′-RACE, and liquid chromatography coupled with tandem mass spectrometry (LC-MS) data to the 4-megabase *C. crescentus* genome. We mapped transcript units at single base-pair resolution using RNA-seq together with global 5′-RACE. Additionally, using ribosome profiling and LC-MS, we mapped translation start sites and coding regions with near complete coverage. We found most start codons lacked corresponding Shine-Dalgarno sites although ribosomes were observed to pause at internal Shine-Dalgarno sites within the coding DNA sequence (CDS). These data suggest a more prevalent use of the Shine-Dalgarno sequence for ribosome pausing rather than translation initiation in *C. crescentus*. Overall 19% of the transcribed and translated genomic elements were newly identified or significantly improved by this approach, providing a valuable genomic resource to elucidate the complete *C. crescentus* genetic circuitry that controls asymmetric cell division.

## Author Summary

*Caulobacter crescentus* is a model system for studying asymmetric cell division, a fundamental process that, through differential gene expression in the two daughter cells, enables the generation of cells with different fates. To explore how the genome directs and maintains asymmetry upon cell division, we performed a coordinated analysis of multiple genomic and proteomic datasets to identify the RNA and protein coding features in the *C. crescentus* genome. Our integrated analysis identifies many new genetic regulatory elements, adding significant regulatory complexity to the *C. crescentus* genome. Surprisingly, 75.4% of protein coding genes lack a canonical translation initiation sequence motif (the Shine-Dalgarno site) which hybridizes to the 3′ end of the ribosomal RNA allowing translation initiation. We find Shine-Dalgarno sites primarily inside of genes where they cause translating ribosomes to pause, possibly allowing nascent proteins to correctly fold. With our detailed map of genomic transcription and translation elements, a systems view of the genetic network that controls asymmetric cell division is within reach.

## Introduction

The *C. crescentus* genome encodes instructions to perform asymmetric cell division using a genetic circuit that integrates transcriptional control from differential chromosome methylation, activation of transcription factors by phosphosignaling pathways, specific proteolysis events, and the subcellular localization of regulatory proteins [1]. Multiple cell cycle events are coordinated with the replication and segregation of the chromosome once and only once per cell cycle [2]. While the *C. crescentus* genome was sequenced 13 years ago [3], our understanding of the transcribed and translated elements in the genome is far from complete.

Tiling arrays have previously been used to map 27 ncRNAs and 769 transcription start sites (TSSs) in the *C. crescentus* genome [4],[5]. Now, using RNA sequencing one can identify transcript architectures at single base-pair resolution and with genome-wide coverage [6]. Recently, global identification of 5′ PPP sites of transcription initiation in the genome using a modified global RACE approach enabled mapping of 2726 TSSs in the *C. crescentus* genome (Zhou *et al.* [unpublished data]). Liquid chromatography-mass spectrometry (LC-MS) based proteomics methods have identified peptides in 66% of annotated coding DNA sequences (CDSs) [7], but poor peptide coverage severely limits mapping of entire CDSs. However, with ribosome profiling, which maps translating ribosomes [8],[9], we have successfully mapped the *C. crescentus* CDSs genome-wide.

We report application of a multi-omic approach utilizing RNA-seq, global 5′-RACE, LC-MS proteomics, and ribosome profiling data sets to identify the RNA transcripts and CDSs in the *C. crescentus* genome at high resolution. We identified transcription units at single nucleotide resolution, 5′ and 3′ UTRs, and the position of all translated CDSs at near single codon resolution in the *C. crescentus* genome. Integration of these datasets allowed the identification of 375 leaderless mRNAs, 94 new small open reading frames, and 106 new noncoding RNAs. Additionally, we mapped 3235 CDSs in the *C. crescentus* genome transcribed from 2201 mRNA transcripts. Our integrated analysis also identifies a plethora of genetic regulatory elements, significantly increasing the knowledge of regulatory complexity encoded by the *C. crescentus* genome. With the identification of the genomic transcription and translation elements, a systems map of the genetic network that controls asymmetric cell division is within reach.

Analysis of the translation initiation sites shows that a majority (75.4%) initiate without a Shine-Dalgarno sequence. A majority of Shine-Dalgarno sites are encoded within the CDSs and, as with *E. coli* and *B. subtilis*, these Shine-Dalgarno sites correlate with pauses in translation elongation [10]–[12]. This suggests that *C. crescentus* uses the Shine-Dalgarno site more commonly for ribosome pausing rather than translation initiation. As suggested from a multitude of predicted bacterial genome annotations [13]–[16], our genomic map provides further experimental evidence that the Shine-Dalgarno-based translation initiation model is not applicable to all bacteria.

## Results

### A multi-level genome-wide gene expression map

**Figure 1:**
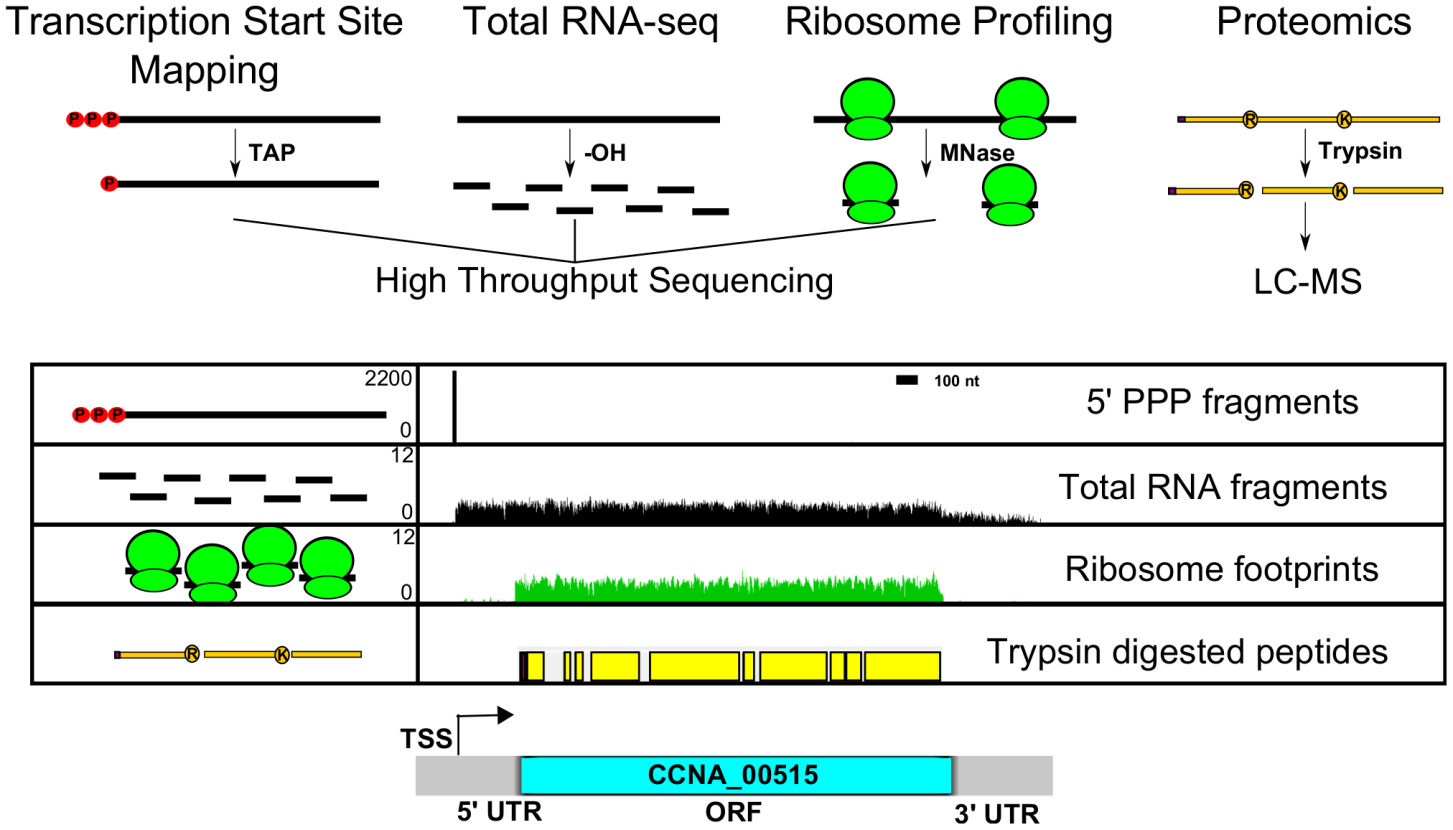
Genome-wide data set integration to map the genetic elements in the *C. crescentus* genome. 5′PPP transcription start site (TSS) (Zhou *et al.* [unpublished data]) (red spheres with black bar), RNA-seq density (black bars), ribosome footprints (green ribosomes), and LC-MS peptide coverage [7] (yellow bars) shown for a single gene (*CCNA_00515*) between 528700 and 532200 bp. 5′PPP data generated from Tobacco Acid Pyrophosphate (TAP) enriched 5′ global RACE. 5′ PPP fragments plotted with Y-axis scale in #reads. Base hydrolyzed (-OH) RNA-seq data plotted with Y-axis scale in log(#reads+1). Micrococcal nuclease (MNase) protected ribosome footprints plotted with Y-axis scale in log(#reads+1). LC-MS-identified tryptic peptides are mapped onto their respective positions of the CDS with potential ribosomal initiated N-terminal residue in purple. Respective genomic features are highlighted including transcriptional start site (TSS), 5′ untranslated region (UTR), coding region (blue bar), and 3′ UTR of the expressed element. Y-axis scales are similar in all subsequent figures.

We integrated multiple *C. crescentus* genomic datasets to map global gene expression features at base-pair resolution (Figure 1). We used a genomic RACE dataset that mapped 2726 TSSs in minimal defined medium allowing promoter and 5′ end RNA identification (Zhou *et al.* [unpublished data] NCBI GEO accession number GSE57366). Additionally, we used RNA-seq data derived from base-hydrolyzed RNA fragments from complex and minimal defined medium to find both the stable 5′ end of the transcript and the length of the transcript onto which we mapped the individual CDSs. To identify translated CDSs, we used both genome coverage of trypsin-digested peptides (identified for 2559 annotated CDSs) in minimal medium during log growth and starvation [7] and ribosome profiling data. Ribosome profiling data were collected from mid-log phase *C. crescentus* NA1000 cultures grown in complex (peptone-yeast extract; PYE) and minimal defined (M2 glucose; M2G) medium. Translation was arrested with 100 µg/mL chloramphenicol, polysomes were digested with micrococcal nuclease, and ribosome-protected mRNA fragments were purified on a sucrose gradient and prepared for high throughput sequencing (Figure S1) [8],[17]. Although the extent of peptide coverage within the CDS was not consistent due to the non-uniform distribution of trypsin cut sites, the ribosome profiling data allowed us to map the expressed CDSs in the genome with high coverage and resolution. The 5′ and 3′ UTRs of the transcript can thus be identified. With this approach we have now identified the global transcript and CDS architecture of the *C. crescentus* genome under the specified growth conditions. Our updated version of the *C. crescentus* genome annotation can be downloaded here (Dataset S1), and has been incorporated in the NCBI NA1000 annotation (accession CP001340).

We identified differentially expressed genes by comparing RNA-seq levels between M2G and PYE medium, and these results agree well with previous microarray measurements (Dataset S1) [18]. In addition, we find that the ribosome profiling levels correlate with the relative amount of protein present in the cell, validating that the ribosome profiling assay is measuring protein production (Fig. S15). The ribosome profiling data also revealed additional changes in translation between growth conditions (Dataset S1). We found 39 genes that are differentially translated with a >2-fold change in translation efficiency (as defined in the Methods) between M2G and PYE medium (Dataset S1). The largest class of differentially translated genes includes eight genes involved in amino acid catabolism. These genes are repressed in M2G, likely due to the absence of amino acids in the medium.

### The *C. crescentus* CDS map

We mapped the CDSs in the *C. crescentus* genome using both LC-MS peptide coverage [7] and ribosome profiling. We initially used the LC-MS peptide coverage and the specificity of trypsin protease to map start codons. Since trypsin cuts proteins after Arg or Lys residues, we identified translation start sites as N-terminal sites not preceded by Arg or Lys codons. To avoid false signals from peptides generated from protein degradation we searched for peptides >20 amino acids, thereby omitting products from the major protease ClpP [19]. A majority of the remaining peptides mapped to ATG, GTG, or TTG start codons or the next codon that would result from cleavage of fMet. In this manner, we identified 621 start codons out of the 3818 annotated CDSs in the NA1000 (CP001340) genome. The remainder could not be identified due to the poor intra-CDS coverage of peptides. Since the ribosome profiling read density matched the 621 verified start codons remarkably well (Figure S2), we used the ribosome profiling data to map all start codons. Importantly, ribosome profiling relies on sequencing the protected mRNA fragment from actively translating ribosomes; thus, the ribosome profiling results can be used to globally map start codons at near complete coverage. Using the density of ribosomes along CDSs, we searched for start codons in the predicted annotation (CP001340) by looking for a continual density of ribosomes from the stop codon to the furthest upstream in-frame start codon. If a peptide was found in the LC-MS data, we refined the search for the start codon from the most N-terminal codon of the peptide to the furthest upstream in-frame start codon covered by ribosome footprints. Additionally, we found many LC-MS peptides and ribosomes positioned outside of annotated CDSs either within intergenic regions or on the opposite strand of hypothetical CDSs. We manually curated these regions to identify the boundaries of the corresponding CDS. Using this multipronged approach we mapped 3235 CDSs in the *C. crescentus* genome. The average increase in the density of ribosomes at the start codon (Figure S2) aided the detection of start codons and, despite heterogeneity in mRNA footprint sizes, allowed us to identify start codons at near single codon resolution.

**Figure 2:**
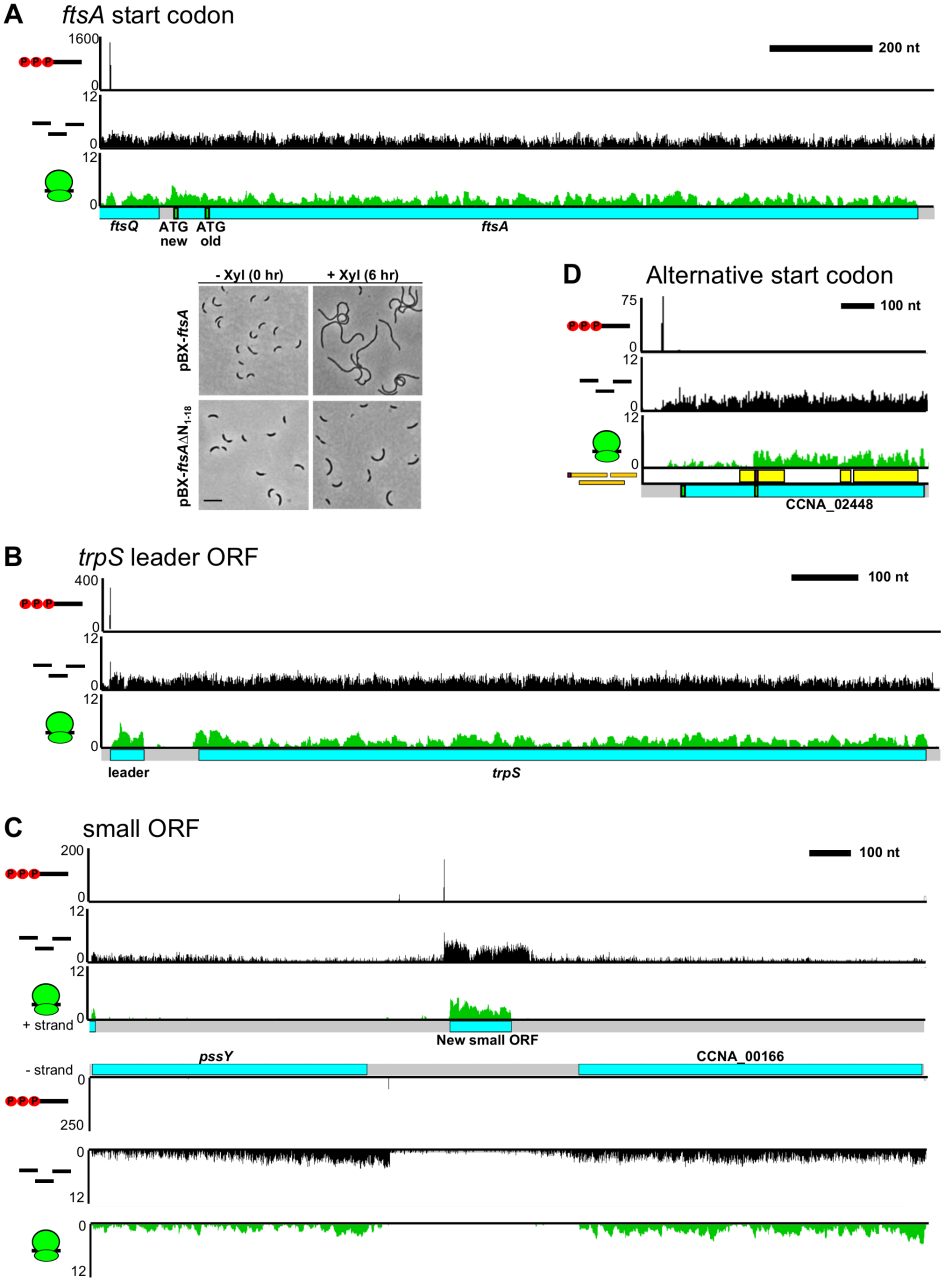
Mapping the *C. crescentus* coding DNA sequence architecture. **A.** Mapping of the correct start codon for ftsA. Shown below are phase contrast images of cells containing a high copy plasmid with either the newly identified start codon (pBX*ftsA*) or the old start codon (pBX-*ftsA*Δ_N1-18_) grown in M2G before and after induction with xylose for 6 hours. Scale bar is 6.05 µm. **B.** Putative small leader CDS identified on the *trpS* mRNA. **C.** Ribosome profiling identification of an intergenic small CDS. **D.** Alternative translation initiation site identified in the *CCNA_02448* mRNA allows translation of two in-frame protein isoforms. Internal start codon was verified by LC-MS.

While 74.3% of the start codons identified were ATG, many CDSs initiate with GTG (14.5%), TTG (10.3%), and a few with CTG (0.7%) (Dataset S1). We also observed a small number of CDSs that begin with other potential near-cognate start codons (0.25%), including one double mismatch GTC codon verified by LC-MS (Dataset S1). In total, we corrected the start codons of 12.8% of annotated CDSs (or 15.7% of those that were mapped), including many that were previously reported to be misannotated or involved in cell cycle regulation including *gcrA*, *chpT*, *sciP*, *sidA*, *divJ*, *parB*, and *ftsA* (Dataset S1) [20]–[22]. We verified that the *ftsA* start codon is 18 codons upstream using western blots (Figure 2A, Figure S11) and found that overexpression from a high-copy plasmid containing the correct start codon yielded a strong cell division phenotype while that of the previously annotated form lacking the N-terminal 18 amino acids ([23]–[25]) causes a less severe phenotype (Figure 2A) even after 24 hours of overexpression (Figure S16), suggesting these 18 N-terminal amino acids are likely functional. In general, predicted start codons are further upstream than our experimentally determined start codons due to the biases of start codon prediction algorithms to pick longer CDSs. However, we identified 69 CDSs with start codons further upstream than the original annotation. We also identified 94 previously unidentified CDSs, most of which encode small proteins of less than 100 amino acids. Some of these small CDSs appear to be leader peptides, such as the small CDS positioned in front of the *trpS* gene (Figure 2B) [26]. It is likely that some of these small leader CDSs have a regulatory role in the expression of their downstream genes [26]. Additionally, we found that 62% of small CDSs are not encoded in the same direction as the downstream genes, indicating that they are not leader peptides and instead likely encode functional proteins (Figure 2C). As tracking the ribosome profiling footprint density allowed us to globally map CDSs in *C. crescentus*, we analyzed the *E. coli* and *B. subtilis* ribosome profiling datasets [10] alone and identified 53 and 70 putative changes to the CDSs in each respective genome (Dataset S6). Finally, we observed cases where a single mRNA has multiple start codons that initiate different isoforms of the protein (Figure 2D) [9]. We identified 75 alternative start codons in the *C. crescentus* genome by searching for internal peptides with N-terminal residues mapping to non-typsin digested ATG sites (Dataset S1).

### Revisiting the role of the Shine-Dalgarno sequence

**Figure 3:**
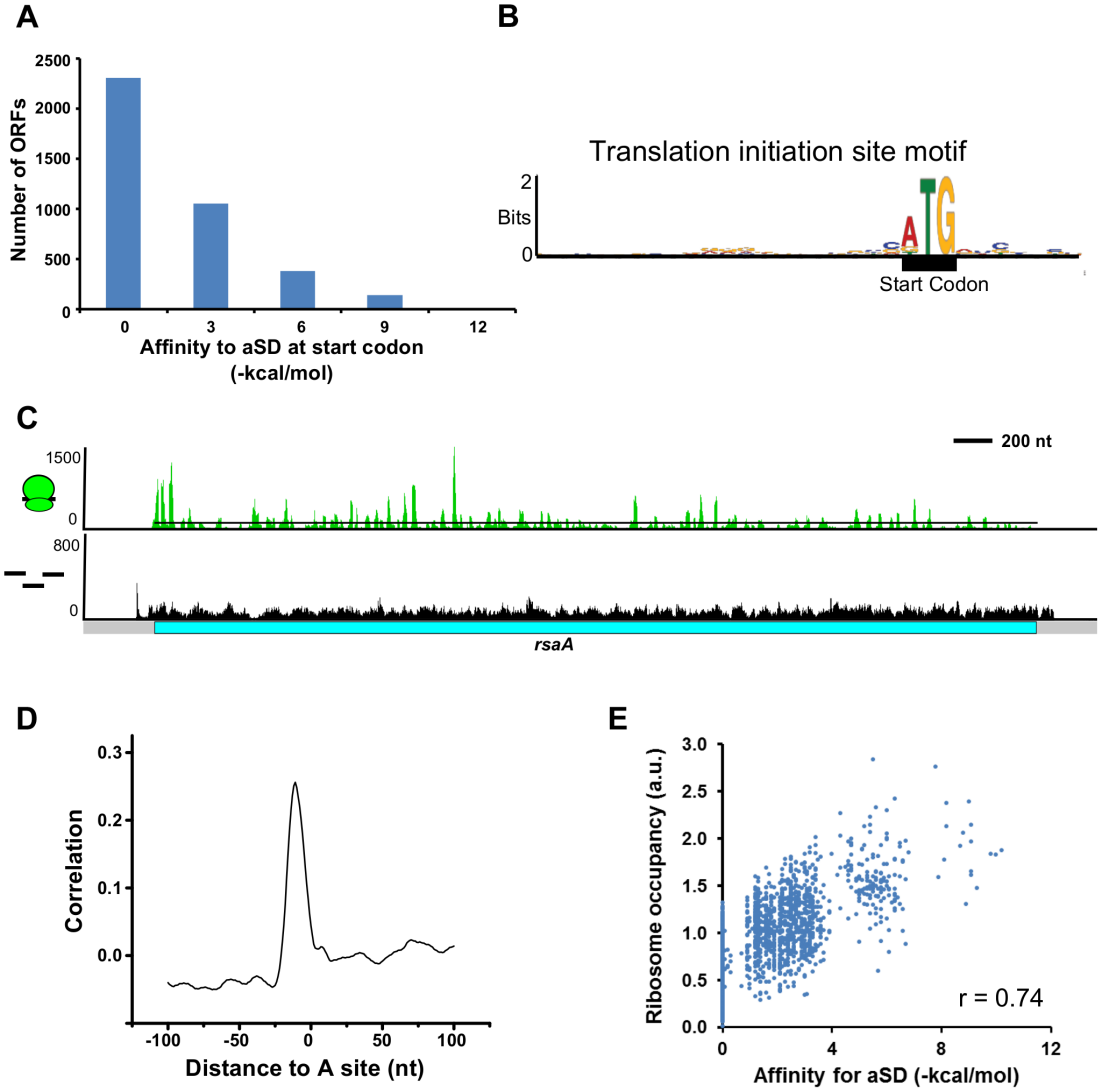
Role of the Shine-Dalgarno sequence in translation initiation and pausing. **A.** Global lack of SD sites in front of start codons. mRNA affinity to the aSD site on the ribosome was calculated using the Free2bind package [16]. −4.4 kcal/mol is the cutoff for SD identification based on the predicted annealing between the aSD and translation initiation region as in [13]. **B.** Translation initiation site motif derived from all start codons in the genome generated in MEME [77]. **C.** Ribosome occupancy profiles reveal pausing identified by peaks of ribosomes above the average read density (black line). Stronger pauses are shown by a larger peak height. Y-axis value is #reads on linear scale. **D.** Plot of the normalized cross-correlation function between pauses in the ribosome occupancy profiles and the presence of SD sequences. The plot is centered at the A-site of ribosome pauses and the peak of correlation occurs in proximity to the aSD site on the ribosome. **E.** Plot of SD binding affinity for the aSD compared to the occupancy of ribosomes translating them. r is the correlation coefficient.

Despite a conserved 3′ end of the rRNA anti-Shine-Dalgarno (aSD) sequence (CCUCC) in all bacteria, only 24.6% (957) of *C. crescentus* CDSs contain a Shine-Dalgarno (SD) sequence in the translation initiation site as determined by the predicted **Δ**G° of annealing between the aSD with the mRNA (Figure 3A, Figure S3A) [16]. While the *C. crescentus* genome is GC-rich (67.17%), the random chance of finding a SD sequence in a translation initiation region is 19.2%, suggesting only slight enrichment of SD sites. The *C. crescentus* translation initiation site motif contains little or no consensus information other than the start codon (Figure 3B). Globally, the predicted RNA stability at the translation initiation site revealed it to be less stable than other regions of the mRNA (Figure S3B), consistent with the model that an unstructured region at the translation initiation site is required to translate mRNAs without a SD sequence at the initiation site [27]. On average we observe a peak of ribosome density at the start codon and a peak, albeit smaller, at the stop codon, suggesting that initiation and termination may be slow steps in *C. crescentus* translation (Figure S4A). However, as the ribosomes were arrested with chloramphenicol, which blocks elongation but not initiation of translation, the enrichment observed at the start codon may not accurately reflect the natural abundance of initiating ribosomes. The ribosome occupancy along genes has peaks along the coding sequence caused by pausing of elongating ribosomes (Figure 3C). As observed in *E. coli* and *B. subtilis* [10], many of the internal pauses in translation elongation appear not to be driven by codon usage (Figure S4B), but instead correlate with internal SD sites in the mRNA coding sequence that base-pair with the 3′ end of the rRNA, stalling ribosome movement (Figure 3D) [10]. The aSD binding strength for the SD sequences correlates with the ribosome occupancy, suggesting that the annealing of the rRNA to the mRNA slows translocation of elongating ribosomes (Figure 3E, Figure S14). These results support the hypothesis that internal SD sites provide a conserved pausing mechanism for bacterial ribosomes even in a genome that has high GC content where SD sequences are more abundant. In accordance with a more prevalent role of the SD in elongation, we see poor correlation with the translation efficiency of mRNAs and the aSD binding strength of their SD sequence at the start codon (Figure S5).

### The *C. crescentus* transcribed RNA map

**Figure 4:**
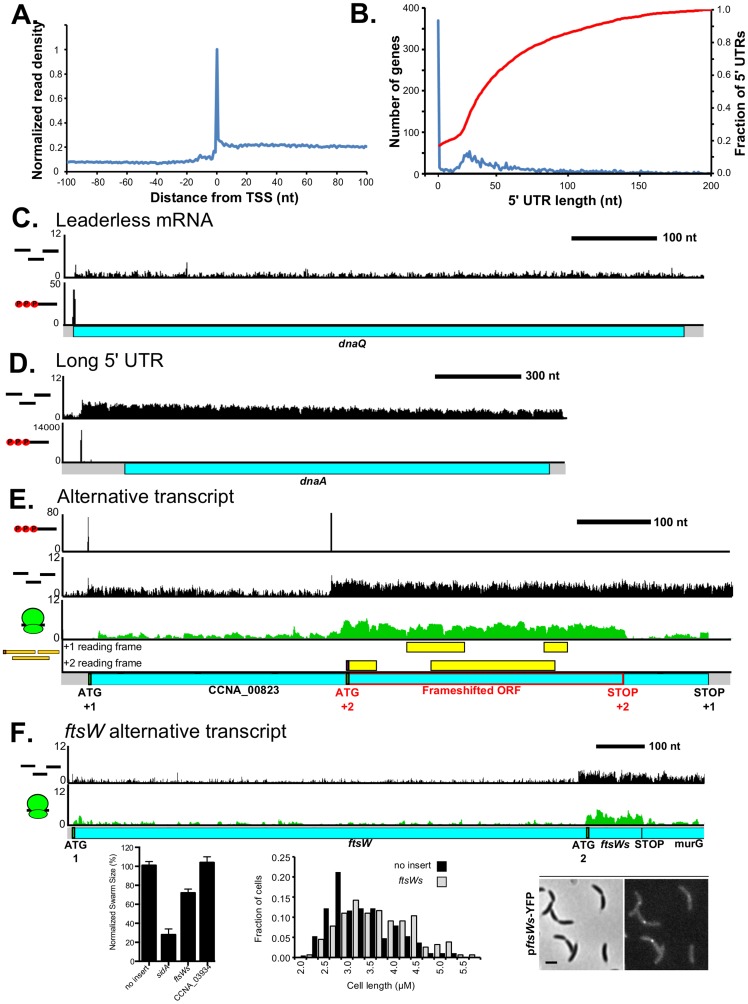
Transcription start site and RNA-seq-derived transcript architecture reveals mRNA complexity. **A.** Metagene plot of the normalized RNA-seq reads centered on the 5′ PPP sites identified by 5′ global RACE (Zhou *et al.* [unpublished data]). RNA-seq reads are mapped to the 5′ nucleotide with an enriched peak resulting from partial shearing of the RNA [70]. **B.** Global distribution of 5′ UTR lengths for all *C. crescentus* mRNAs with identified 5′ ends shown in blue with Y-axis scale on the left. Cumulative distribution of 5′ UTRs less than 200 nt shown in red with Y-axis scale the right. **C.** Leaderless *dnaQ* mRNA, where transcription is initiated at the 5′ nucleotide of the initiating ATG. **D.** Long 5′ UTR (150 nt) of the *dnaA* mRNA. **E.** TSS selection yields alternative translation products: A full length CDS is translated in the +1 reading frame measuring 804 nt. An internally initiated transcript encodes a 360 nt CDS that is translated in the +2 reading frame highlighted in red. LC-MS peptides corresponding to both CDSs are shown in yellow. **F.** Alternative transcripts drive two different start codons for *ftsW* with the position of each translation start site (initiating ATG codon) marked below. ATG 2 is in the same reading frame as ATG 1. Shown on the left is a low agar swarmer plate assay for motility and cell division defects, with the cell division inhibitor *sidA* as a positive control and the *ileS* leader CDS (CCNA_03934) as a negative control. Shown in the middle is a cell length distribution from cells containing a xylose inducible high-copy plasmid (pBXSPA) with *ftsWs* or no insert. Shown on the right is the localization of *ftsWs*-YFP expressed from a low copy plasmid p*ftsW*s-YFP where the promoter for *ftsW* has been replaced by two transcription terminators.

To identify the RNA transcript units we used a global RACE dataset that maps 5′ PPP-sites of transcription initiation (Zhou *et al.* [unpublished data]) together with RNA-seq density measured here. We found good overlap of the TSSs between the datasets. When the RNA-seq density is centered at the TSSs identified by 5′ global RACE, we observed an increase in RNA-seq read density at the same 5′ nucleotide (Figure 4A). By comparing the RNA-seq data to the TSSs we were able to map the length of the major form of the transcriptional unit and in some cases where an internal TSS exists, allowing us to identify potential isoforms of transcripts. The transcripts mapped by our RNA-seq approach agree well with published northern blots (Dataset S7). In addition, by comparing the transcript unit with the mapped CDSs, we were also able to determine which RNAs encode proteins under the growth conditions tested. In the RNA-seq data sets we found 74% of reads map within CDSs, 21% of reads map to intergenic regions, and 5% map antisense to CDSs. In total, 96.2% of the genome was transcribed among the conditions tested. Together, these data now provide a comprehensive map and functional classification (coding or noncoding) of the expressed RNAs in the *C. crescentus* genome with single base-pair resolution.

### mRNA architecture

The global distribution of mRNA leader lengths in *C. crescentus* (Figure 4B) shows that 57% of 5′ UTRs are between 15 and 60 nt with some spanning >100 nt. Surprisingly, we observed 375 leaderless mRNAs (9.6% of the cell's CDSs) (Figure 4C). The 5′ nucleotide is the first base of the start codon in a leaderless mRNA that is able to initiate translation on bacterial, archaeal, and eukaryotic ribosomes, suggesting it is an ancient mechanism of translation initiation [28]. Leaderless mRNAs have been found to be rare in most bacteria [29]–[31] and previously only two leaderless mRNAs were identified in *C. crescentus*: *dnaX* and *hemE* [32]. The presence of many leaderless mRNAs in *C. crescentus* and 171 in *S. meliloti* [33] suggests translation of leaderless mRNAs may occur more commonly in the alpha-proteobacteria than previously anticipated.

In contrast to leaderless mRNAs, we identified 286 mRNAs that have long 5′ UTRs >100 nt (Figure 4D), which may play regulatory roles in translation. For example, *dnaA* mRNA encodes a 155 nt 5′ UTR that contributes to the repression of translation, suggesting the 5′ UTR helps regulate the level of the protein needed for proper cell cycle regulation [34]. Additionally, four 5′ UTRs appear to encode conserved riboswitches that are capable of regulating the expression of downstream genes upon direct metabolite binding to the RNA [35] (Table S1).

We also observed genes for which the mRNA is transcribed from an internal site driving translation of an alternative translation initiation site. For example, *CCNA_00832* has an internal TSS which is translated from a start codon in the +2 reading frame, resulting in a distinct protein compared to the lowly expressed full length mRNA isoform (Figure 4E). In addition, we find that the cell division gene *ftsW* has two mRNA isoforms which result in translation of two different length proteins in the same reading frame, with the smaller form (*ftsWs*) being more highly translated (Figure 4F). No PPP site was identified for the small form of the mRNA; however, it does contain a good sigma 70 site [36] 35 nt upstream of the internal 5′ end. Additionally, when *ftsW* was inserted into a low copy plasmid lacking the promoter for the full length *ftsW* CDS, we observed accumulation of FtsWs protein (Figure S11) suggesting it is transcribed from an internal transcript. Overexpression of *ftsWs* causes a marked motility and cell division phenotype in a low-agar swarming plate assay as well as an increase in cell length when grown in liquid culture (Figure 4F, S6). Despite the small size of this 35 amino acid isoform, FtsWs can localize to sites of constriction (Figure 4F) suggesting it may play a role in cell division. Altogether, we observe that alternative transcripts can drive alternative translation products increasing the diversity of proteins encoded in the genome.

### Non-coding RNA architecture

**Figure 5:**
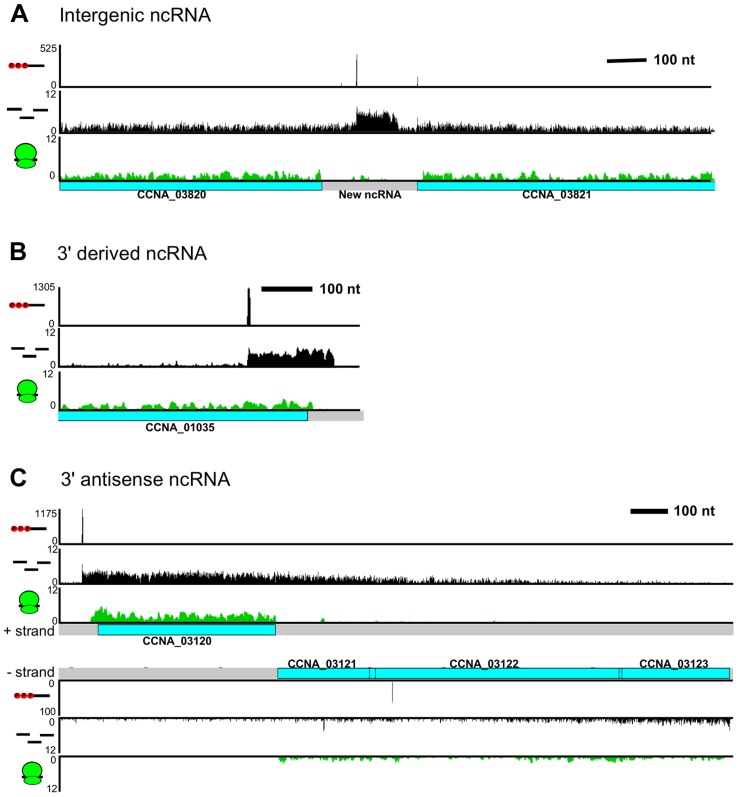
*C. crescentus* noncoding RNA architecture. **A.** Previously unannotated intergenic small RNA. **B.** Small non-coding RNA with a TSS encoded within the 3′ region of *CCNA_01035*. **C.** *CCNA_03120* mRNA with an extended 3′ UTR overlapping the *CCNA_03121-3* operon on the antisense strand.

We observed 133 non-coding RNAs (ncRNAs), adding 106 new ncRNAs to the 27 previously identified using tiling arrays [4] (not including conserved ncRNAs such as tRNAs, rRNAs, RNaseP, 6S RNA, 4.5S RNA, and tmRNA). Most of the ncRNAs are expressed from intergenic regions (Figure 5A) and ribosome profiling data showed that these regions are not translated. Some ncRNAs are transcribed from TSSs in the 3′ end of a CDS (Figure 5B), which, similar to *Salmonella*, allows the 3′ UTR regions to act as a reservoir for ncRNAs [37].

RNA-seq data showed widespread antisense RNA transcribed throughout the *C. crescentus* genome accounting for 5% of non-tRNA/rRNA reads. Global RACE 5′ PPP mapping revealed that antisense TSSs are found within 15% of CDSs (Zhou *et al.* [unpublished data]). We observed that the 3′ UTR of an mRNA can extend into the coding regions of downstream genes oriented in the opposite direction forming a long antisense RNA with respect to the mRNAs of these downstream genes (Figure 5C). We found overlaps extending over up to three genes. For example, the 3′ UTR of *CCNA_03120*, a gene predicted to encode a protein involved in chemotaxis, extends into the coding regions of an operon containing genes *CCNA_03121*, *CCNA_03122* (putative integral membrane protein), and *CCNA_03123* (metal regulated homo-dimeric repressor).

### Complexity in the architecture of operons

**Figure 6:**
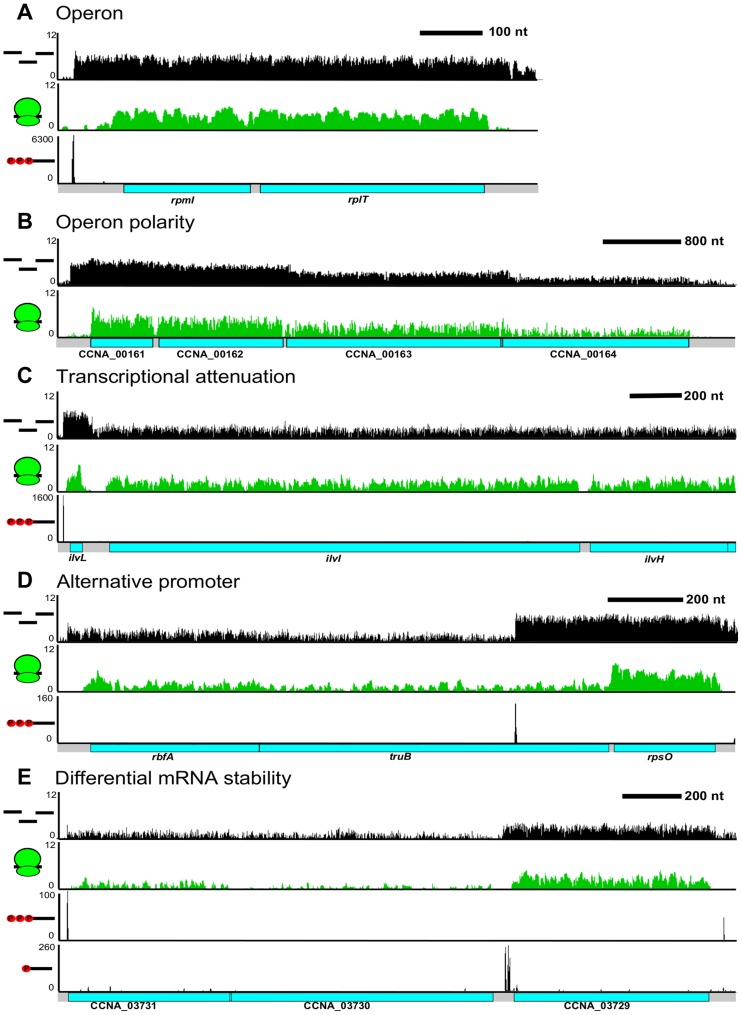
Complex regulation of *C. crescentus* operons. **A.** Classical ribosomal protein operon containing *rpmI* & *rplT*. **B.** Polarity with decreased RNA read density at the 3′ CDSs in the *CCNA_00161-4* operon. **C.** Transcription attenuation through the *ivlL* leader CDS to regulate expression of the *ilvIH* operon. **D.** Alternative TSS in the *rbfA truB rpsO* operon can drive differential CDS expression. **E.** Potential operon cleavage site between *CCNA_03729* and *CCNA_03730* by the presence of a 5′ monophosphate on the RNA. The higher RNA stability the *CCNA_03729* RNA can allow higher 3′ gene expression levels.

The operon has been traditionally defined as a single co-transcribed unit that yields a single polycistronic mRNA. Using our CDS and RNA maps, we were able to identify operons as mRNAs with >1 CDS (Figure 6A). We observe 863 operons in the *C. crescentus* genome encoding 65% of all CDSs in the genome. We found that 55% of operons contain 2 CDSs (Figure S7); however, a few operons are quite large with up to 29 CDSs in a single operon. Examples of these include the type IV pilus operon (12 CDSs) [38], one of the ribosomal protein operons (24 CDSs), and the NADH dehydrogenase operon (29 CDSs), the largest *C. crescentus* operon. The distribution of operon sizes for *C. crescentus* is similar to that for *M. pneumonia* [39], *H. pylori* [31], and *E. coli* [40]. In many operons, such as those of ribosomal proteins (Figure 6A), the expression level of each CDS is similar yielding the proper stoichiometry of ribosomal proteins of one per ribosome. However, we find that many *C. crescentus* operons do not have equal expression of the encoded CDSs at the RNA and translation levels (Figure S8).

Different levels of expression of RNA for contiguous CDSs within a single operon can be caused by a multitude of factors. The most well characterized mechanism is transcriptional polarity, driven by translation rate, transcription elongation factors, and/or termination factors to cause the 3′ end genes to have reduced levels of expression (Figure 6B). Additionally, operons can be regulated by transcriptional attenuators that down-regulate transcription of the trailing genes (Figure 6C). As shown originally in the *E. coli trp* operon [26], the *ilvBN* operon leader has tandem Ile and Val codons which, upon conditions of low tRNA^Ile^ and tRNA^Val^ aminoacylation, cause ribosome pausing at these codons, blocking a rho-independent terminator hairpin from forming and allowing RNA-polymerase to elongate through the *ilvBN* operon [41]. Uneven expression can also cause the 3′ end CDSs to be expressed higher than 5′ end CDSs. We observed that 349 operons contained an alternative TSS (Figure 6D) that could potentially drive higher expression of downstream CDSs. Expression from these internal TSSs was observed to be dynamically regulated during the cell cycle (Zhou *et al.* [unpublished data]). We also observed operons that appear to have 3′ end genes whose mRNAs are more stable (Figure 6E). In these cases, the operon has only a single TSS and contains a downstream 5′ P site, indicative of an RNaseE cut site. Since the last CDS has a higher mRNA level, it is likely that the 5′ end of the transcript is less stable. Altogether, 64% of the operons appear to have a >2 fold change in RNA level among different CDSs suggesting that most operons are regulated co- and post-transcriptionally to ensure appropriate RNA levels of each encoded CDS.

Operons appear to be highly regulated by having both multiple TSSs and different transcription termination sites. We therefore calculated the total number of TSSs per operon and found that *C. crescentus* operons have an average of 1.3 TSSs per operon driving multiple mRNA isoforms. Additionally, the number of operons that have successive ≥5-fold drops in RNA level between encoded CDSs is 125, suggesting that polarity of operons also drives many isoforms. In total, we estimate that *C. crescentus* operons have an average of 1.5 isoforms per operon generated either from alternative TSSs or polarity and 0.5 *cis*-encoded regulatory features including antisense RNAs, riboswitches, and transcription attenuators. The high number of isoforms and regulatory features suggests that operons can be highly regulated at the transcription and RNA levels. Together with the 75 CDSs that can be initiated internally to drive different protein isoforms, this suggests that the *C. crescentus* genome contains significant regulatory complexity.

## Discussion

### Integration of genomic datasets reveals regulatory complexity

We used multiple datasets from ribosome profiling, RNA-seq, 5′ global RACE, and LC-MS [7] to identify and quantify the transcribed and translated elements of the *C. crescentus* genome with high resolution and near complete coverage (Table 1). Ribosome profiling provides a way to map CDSs that greatly surpasses LC-MS in coverage. We found misannotation of the start codons of many important genes involved in the *C. crescentus* cell cycle (Dataset S1), including the essential cell division gene *ftsA* (Fig. 2A, Fig. S11), and found that the truncation of the N-terminal 18 amino acids absent in the previously annotated start codon affects the function of FtsA. We found two cases where previously predicted ncRNAs [4] are, in fact, translated (Dataset S1). Additionally, ribosome profiling identified 94 previously unknown CDSs, a majority of which are <50 amino acids. In total we observe 94 small CDSs of <50 amino acids in the genome. The role of these small proteins in *C. crescentus* is largely unexplored; however, small proteins have been reported to have important functions in *B. subtilis*, *E. coli*, and eukaryotes [42],[43]. A recent identification of a small protein in *C. crescentus* that can delay cell division upon DNA damage suggests this class of proteins indeed can perform important cellular functions in *C. crescentus* [20].

**Table 1:** Genome annotation summary.

Total number of CDSs mapped	3235
Number of CDSs with corrected start codons	529
New CDSs	94
Deleted CDSs	28
New ncRNAs	106
Leaderless mRNAs	375

We discovered 106 new ncRNAs in the *C. crescentus* genome that are expressed during normal growth. However, most of the identified *C. crescentus* ncRNAs are not conserved in other genomes outside of the *Caulobacteraceae* with only a few present in other alpha-proteobacteria. The function of only one of these ncRNAs in *C. crescentus* has been characterized, *crfA*, which was shown to be involved in the response to carbon starvation [4],[44]. In other bacteria, small ncRNAs have a variety of functions, but most commonly they are involved in annealing to mRNAs with complementary sequences and regulating translation or mRNA stability [45]. Most ncRNAs identified in bacteria function through the RNA chaperone *Hfq* [45],[46]. *Hfq* is thought to both stabilize the ncRNA and facilitate annealing between the ncRNA and the target mRNAs. In *C. crescentus* the ncRNA substrates of *Hfq* have not been identified; however, *Hfq* was found to be non-disruptable in a high-throughput transposon mutagenesis screen [22] suggesting an important role for ncRNA regulation. Additionally, 14 of the *C. crescentus* ncRNAs are cell cycle regulated (Zhou *et al.* [unpublished data]), suggesting these ncRNAs may play a role in cell cycle progression.

With our CDS mapping approach we identified upstream leader peptides and alternative start codons (Figure 2A,B,D). While translation of upstream leader peptides can often regulate expression of the downstream gene, it is possible that these CDSs may also produce functional proteins. Alternative start codon selection in eukaryotes has been shown in some cases to control subcellular localization and to cause functional switches in proteins by translating forms lacking functional domains [47]–[49]. The cell division gene *ftsW* is made in a full length and short form (Figure 4F), both of which can localize to the site of constriction at the midcell (Figure 4F) [25]. Overexpression of *ftsWs*, the short form of *ftsW*, gave rise to a motility and cell division defect in the swarmer plate assay, leading to a modest elongation of the cells (Figure 4F, S6). As the mRNA for the full length *ftsW* is activated in the late predivisional cell, it will be important to measure the cell cycle-regulated translation of both the *ftsW* long and *ftsWs* short forms to understand their roles in regulating cell division.

The vast amount of regulatory RNA elements identified by this approach suggests that there's an unexplored level of cell cycle gene expression control that remains to be investigated. Indeed, as seen in other bacteria, the examination of RNA levels in operons suggests that most operons are not consistent with the classical model of one polycistronic transcriptional unit, suggesting that regulation of operons is more complex [31],[39],[50]. In support of this we estimate that on average, each mRNA and operon contains 2.0 cis-encoded regulatory features (alternative TSS, antisense RNA, internal TSS, internal start codon, lower 3′ RNA density) suggesting combinatorial regulation. Altogether, in the *C. crescentus* genome we identified ncRNAs, leaderless mRNAs, alternative translation initiation sites, small upstream CDSs, antisense RNAs, alternative transcription initiation sites, transcriptional polarity of operons, and differential RNA stability of operons. These elements are spread throughout the genome and suggest that co/post-transcriptional regulation is likely an important mechanism for cell cycle regulation of gene expression. In support of this, many antisense RNAs and ncRNAs are differentially activated at specific stages of the cell cycle (Zhou *et al.* [unpublished data]). An important goal will be to understand how the RNA regulatory elements affect cell cycle stage-specific translation and mRNA stability to identify their role in the genetic circuitry that drives the cell cycle.

### Diverse mechanisms of translation initiation

Bacterial translation start site selection is thought to occur by the 30S ribosome subunit binding to the SD site on the mRNA [51],[52], spaced approximately 5 nt away from the start codon [53]. While the kinetic events of translation initiation on SD led mRNAs have been well studied [54], initiation on leaderless mRNAs and non-SD containing mRNAs are less well understood. Recent reports suggest that non-SD led mRNAs have an unstructured region at the start codon [27], which was also seen in *C. crescentus* (Figure S3B). Additionally, non-SD led mRNAs may contain motifs that bind to sites on the rRNA outside the aSD region [55],[56]; however, we do not see abundant motifs that can explain initiation (Figure 3B). We observe that around the start codon the predicted mRNA folding stability is lowest, suggesting that having an unstructured region may be vital for non-SD mRNA binding to ribosomes (Figure S3B) [27].

Leaderless mRNAs are initiated by preassembled 70S/80S ribosomes and can be initiated by ribosomes from all three domains of life [28],[57],[58]. We find that leaderless mRNAs have no specific motif for translation initiation, but instead have an unstructured region that is shifted from the start codon further towards the 3′ end of the translation initiation site likely ensuring the AUG is accessible to bind initiator tRNA in the mRNA channel of the ribosome (Figure S3B). The *C. crescentus* genome appears to contain the second highest relative number of leaderless mRNAs of any bacterium characterized to date with 375 in a 4.0 mb genome, only behind *Mycobacterium tuberculosis* with 505 in a 4.4 mb genome [59]. Interestingly, in *C. crescentus* leaderless mRNAs are translated with similar efficiency to mRNAs containing a leader (Figure S10) suggesting *C. crescentus* translation is adapted to use leaderless mRNAs as substrates during normal growth and not with a stress induced mechanism as in *M. tuberculosis* or *E. coli* [59],[60].

Analysis of sequenced bacterial genomes shows an abundance of non-SD led mRNAs across bacteria, suggesting that the SD dominated mechanism, which is abundant in *E. coli* and *B. subtilis* (66.9% and 94.3% of CDSs use a SD sequence, respectively (Figure S9 [16])), is not abundantly used in other bacterial species [13]–[16],[27],[61]. Furthermore, bioinformatics predictions have estimated that the fraction of genes with start codons preceded by SD sites is only 54.3% across bacteria [15]. In *C. crescentus* only 24.6% of all start codons are preceded by a SD sequence, providing direct evidence that SD mediated translation initiation is not the major mechanism. Interestingly, *C. crescentus* ribosomes do not initiate on bacteriophage Ms2 or T4 mRNAs and *E. coli* ribosomes do not initiate on *Caulobacter* phage Cb5 mRNA, suggesting the translation machinery of these bacteria have different specificities for translation initiation sites despite a similar aSD sequence of the rRNA [62],[63]. Overall, this suggests that the low level of SD sites in *C. crescentus* translation initiation sites (24.6%) may be due to an adaptation of *C. crescentus* translation machinery to initiate on non-SD led mRNAs. In support of this, we observe equivalent translation efficiency of leaderless, non-SD led, and SD led mRNAs (Figure S10). Thus, *C. crescentus* provides a useful model system to investigate the molecular mechanisms of translation initiation on both non-SD and leaderless mRNAs.

### Role of the anti-Shine-Dalgarno in pausing

Using our experimentally determined CDS features we found that *C. crescentus* uses SD sites primarily for ribosome pausing within the CDSs instead of for translation initiation. We did not observe that *C. crescentus* ribosomes preferentially paused at rare codons (Figure S4B), similar to *E. coli* and *B. subtilis* when cultured in conditions with sufficient nutrients, but instead at internal SD sites within the mRNA (Figure 3D,E) [10]. Upon starvation of *E. coli* or *B. subtilis* cells for serine, pausing is observed at serine codons [10],[12] suggesting that depleting aminoacyl-tRNA levels can cause significant codon dependent pausing [26],[64]. In *C. crescentus*, as in *E. coli* [10], SD sites are selected against in the CDS (Figure S13), presumably due to their strong ability to pause ribosomes. Indeed, the presence of internal SD sites within CDSs has been shown to cause long pauses in a single molecule ribosome translocation assays [65]. Additionally, ribosome pausing at internal SD sites has also been shown to be an important element for ribosome frame shifting [66],[67] and likely affects other cotranslational processes such as protein folding [68]. The aSD site in the ribosome is conserved across all known bacteria (Figure S12) [14], even in those lacking abundant SD sites at start codons. As *C. crescentus* has evolved to have a larger apparent role of the SD for pausing than initiation, perhaps the strong conservation of the aSD site is due in part to its role in programmed ribosome pausing.

## Materials and Methods

### Ribosome profiling and RNA-seq

*C. crescentus* strain NA1000 was grown in M2G or PYE overnight in 5 mL, transferred to 25 mL and grown overnight, then diluted into 500 mL and grown to an OD600 of 0.5. Cells were treated with 100 µg/mL of chloramphenicol for 2 minutes then harvested by centrifugation and flash frozen in liquid nitrogen. Cells were subjected to mixer milling (6 cycles for 3 min at 15 Hz) while frozen in liquid nitrogen. A small amount of the lysate was saved for RNA-seq and the rest was used for ribosome profiling. Ribosome profiling was performed as in [10],[17]. To prepare the RNA-seq libraries, total RNA was extracted from the frozen cell pellet by hot acid-phenol extraction and RNA integrity was verified on the bioanylzer (Agilent). rRNA was removed by MICROBExpress gram negative rRNA removal kit (Ambion). The resulting RNA was base hydrolyzed at 95°C in alkaline hydrolysis buffer (50 mM sodium carbonate pH 9.2, 1 mM EDTA) for 23 minutes and size selected between 20 and 45 nt on a denaturing PAGE gel (10% acrylamide 1× TBE/7M Urea). Library prep was performed as in [10],[17] for both RNA-seq fragments and ribosome footprints. DNA libraries were sequenced on the Illumina Hiseq 2000 or Genome Analyzer platforms. Ribosome profiling reads were mapped to the NA1000 genome sequence (CP001340) using bowtie 0.12.8 [69] and center weighted as in [10]. RNA-seq reads were mapped to the 5′ nucleotide to find the 5′ ends or to the full read sequence for mapping transcripts. Data for two ribosome profiling and two RNA-seq datasets (one set for both M2G and PYE) were deposited into the gene expression omnibus (accession number GSE54883).

### CDS mapping

Ribosome profiling read density (Datasets S2 & S3) and the LC-MS derived tryptic peptides were both mapped to the NA1000 genome sequence (CP001340). Using the predicted CDS architecture in the annotation file downloaded from genbank (accession number CP001340) we found tryptic peptides in 66% of the CDSs. Tryptic peptides were directly used to map start codons if the N-terminal codon (or the previous codon in the case of formyl-Met processing) mapped to regions where the previous codon was not an Arg or Lys codon. Since the coverage of the tryptic peptides at the start codon is poor we used the ribosome profiling read density to map the remainder of start codons. We defined start codons as the most upstream ATG, GTG, CTG, or TTG codon with >1/20 the ribosome profiling read density. If no 1^st^ position mismatches were found we searched for single position mismatches in the 2^nd^ and 3^rd^ positions. If no single position ATG mismatches were found, we used the resulting codon only if they matched the beginning of ribosome density and contained a LC-MS tryptic peptide not preceded by an Arg or Lys codon. Each potential start codon which fit this criterion was manually annotated to ensure accuracy. If we identified two adjacent potential start codons we selected the most upstream start codon.

To identify new CDSs we searched for intergenic regions of significant ribosome density. We considered a region a CDS if the ribosome density strictly mapped between start codons to stop codons. We also checked for CDSs that had greater antisense than sense ribosome footprints and manually corrected genes predicted on the wrong strand. We deleted hypothetical CDSs that significantly overlapped other CDSs encoded on the opposite strand or that significantly overlapped tRNA genes.

To map CDSs on leaderless mRNAs we found that the center-weighted ribosome footprints often began 12–18 nt after the start codon as the ribosome footprints were shorter. We therefore identified either 5′ PPP ends or the 5′ end of the RNA-seq read density for each potential start codon. If tryptic peptides matched the 5′ end we annotated it as a leaderless mRNA. Alternatively if no tryptic peptide was found, we mapped leaderless mRNAs if the 5′ end matched the first nucleotide of the start codon and the center-weighted ribosome footprints mapped to the 5′ end of the mRNA. We verified this signature on leaderless mRNAs *dnaX* and *hemE* [32].

### Transcript mapping

The 5′ end was mapped based on the increased peak intensity of the RNA-seq data at the 5′ nucleotide resulting from partial shearing of the RNA [70] (from Dataset S4 & S5) and/or presence of a 5′ PPP site. 3′ ends were mapped based on an increased 3′ end peak intensity before a drop in RNA level if present, or estimated based on the drop in RNA reads. Non-coding RNAs were identified by examining intergenic or antisense stretches of RNA-seq density. We considered an RNA non-coding if no CDSs were detected within the transcript boundaries. 5′ UTR length distribution was calculated using mapped 5′ RNA ends identified within 300 nt upstream of the start codons or within the last 30% of the upstream CDS, whichever is the shorter distance. 5′UTRs longer than 300 nt were curated manually. To identify known riboswitch elements we searched the 5′ UTR sequences in the Rfam database.

### Operon identification

Using the predicted NA1000 operon predictions [71] we appended new CDSs to operons using the following criteria: 1) CDSs that overlap or were less than 40 nt away with the upstream operon or CDS were annotated as either part of the previous operon or as a new operon if overlapping with an upstream single CDS. 2) CDSs less than 260 nt from an upstream CDS were manually inspected and annotated. To use the new CDS map to refine operon predictions we split predicted operons at sites between individual CDSs if they met the following criteria: 1) Intergenic region between CDSs must be >40 nt, 2) Reads per nucleotide must be >20, and 3) a >10 fold difference in RNA-seq read density between the CDS and intergenic region must be observed.

### SD site prediction

SD sites were calculated using the Free2Bind package [16]. To identify SD affinity for a translation initiation site, we calculated the annealing affinity of 5′-CACCUCCU-3′ sequence of the rRNA with a 1 nt sliding window from −100 to +100 nt of the translation initiation site. Presence of a SD motif was determined if the lowest predicted ΔG° of annealing between the rRNA and mRNA was less than −4.4 kcal/mol [13] in a window between −20 and −5 nt upstream of the translation initiation site [53]. To estimate the background SD affinity encoded by a random sequence of nucleotides at the GC% of the *C. crescentus* genome we calculated the SD affinity on 10,000 randomized sequences. 19.2% of random sequences contained our criteria for a SD motif.

### Ribosome pausing analysis

Global ribosome pausing analysis was performed as in [10] on genes with average read coverage >10 reads per codon in M2G medium. The average normalized cross-correlation function of sequence elements relative to pause sites was calculated on genes greater than 160 nt long and >10 reads per codon.

### mRNA folding prediction

Predicted ΔG° of RNA structures were calculated using the RNAFold program in the Vienna RNA package [72] as in [27]. The minimum free energy was calculated in a 50 nt sliding window moving in 1 nt increments from 100 nt before to 100 nt after the start codon.

### Calculation of RNA-seq and ribosome profiling expression levels between media

Levels of gene expression were calculated using the reads per kilobase per million mapped reads (R.P.K.M.) [73] between samples. Ribosome profiling data were corrected for initiating and terminating ribosomes by removing the first 10 codons and the last 5 codons from the R.P.K.M. calculation.

After removing genes with less than 30 reads in a given sample, genes were classified as differentially translated between M2G and PYE if they had a greater than 2-fold change in the translation efficiency = 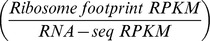.

### Light and fluorescence microscopy

Images were collected as described in [74] on M2G 1.5% agarose pads using a Leica DM6000B microscope. For image analysis MicrobeTracker software [75] was used to determine cell outlines and measure the cell length.

### Swarmer assay

Cells were grown to mid-log phase, normalized to OD600 0.3, and spotted on PYE/0.3%-Bacto-Agar/0.3%-xylose/kanamycin plates. Cells were grown for 2–4 days in a humid 28°C chamber, and imaged on a gel imager. Colony size was calculated using imageJ.

### Western blotting

Whole cell lysates were generated by growing 1 mL cultures to mid-log, resuspending the cells in 1× Laemmli sample buffer, and boiling at 95°C. Lysates were run on TRIS-Gly SDS-PAGE gels (Bio-Rad 4–15% or 10% acrylamide) and transferred to PVDF membranes (Millipore). Immunoblotting was performed using anti-GFP (Roche) or anti-FtsA sera followed by detection using chemiluminescent substrate (PerkinElmer). Band intensity was calculated using ImageJ.

### Strains

A list of all strains can be found in Table 2. To generate all replicating plasmid containing strains, *C. crescentus* NA1000 was transformed with the following plasmids and selected using standard procedures on PYE plates supplemented with antibiotics. All plasmids were sequence verified.

**Table 2:** List of strains used in this study.

Strain	Background	Source
LS101	NA1000	
UJ838	NA1000 ΔclpA	[78]
LS5389	NA1000 Δ*ftsA* pxylX::*ftsA*	[79]
EG083	NA1000 Δ*ftsA* pxylX::ΔN1-18*ftsA*	[25]
LS5364	NA1000 pBX*-ftsA*	[79]
EG023	NA1000 pBX- *ftsA*ΔN1-18	
LS5380	NA1000 p*ftsWs*	This study
LS5381	NA1000 CCNA_03915::YFP	This study
LS5382	NA1000 CCNA_03965::YFP	This study
LS5383	NA1000 CCNA_03904::YFP	This study
LS5384	NA1000 CCNA_03919::YFP	This study
LS4020	NA1000 XylX::YFP	[76]
LS5385	NA1000 pBX-SPA	This study
LS5386	NA1000 pBX*ftsWs*-SPA	This study
LS5387	NA1000 pBX*sidA*-SPA	This study
LS5388	NA1000 pBX*CCNA_03934*-SPA	This study

To generate pBXSPA small CDS overexpression plasmids, an SPA tag was inserted into pBXMCS-2 between the EcoRI and SacI sites. Then, *ftsWs, sidA, and CCNA_03934* were inserted between NdeI and EcoRI.

To generate p*ftsWs*, *ftsW* was inserted into pRVYFPC-6 between HindIII and KpnI, removing any promoter for *ftsW* and blocking full length expression with two 5′ transcription terminators.

To generate YFP integrating strains, 500 bp of DNA upstream of the stop codon was cloned in frame with YFP in pYFPC-4 using Gibson assembly. Resulting integrating plasmids were transformed into NA1000 and selected on PYE gentamycin.

To generate pBX-*ftsA*, *ftsA* was inserted into pBXMCS-2 [76] between the NdeI and PstI sites.

To generate *ftsA::ftsA*Δ*C* P*_xylX_*-*ftsA*, *ftsA*_1–375_ was inserted into pXMCS-2 [76] between the NdeI and PstI sites. The resulting plasmid (pXMCS-2 *ftsAΔC*) was subsequently transformed into NA1000 resulting in a single integration event at the *ftsA* locus that simultaneously truncated the native *ftsA* gene while introducing P*_xyl_-ftsA*. Transformants were selected on PYE kanamycin and xylose.

pBX- *ftsA*ΔN1-18 was a gift from Erin Goley.

## Supporting Information

Figure S1 *C. crescentus* polysome digestion with micrococcal nuclease. Absorbance at 260 nm of polyribosomes separated on a 10 to 55% sucrose gradient before (blue) or after (red) digestion with micrococcal nuclease. After digestion the 70S peak was purified and the resulting mRNA footprints were prepared for high-throughput sequencing.
(TIF)

Figure S2 Metagene plot of ribosome density at start codons. The average ribosome density plotted at CDSs where start codons were verified by LC-MS.
(TIF)

Figure S3 RNA folding energy and Shine-Dalgarno affinity to rRNA for *C. crescentus* translation initiation sites. Blue – SD containing CDSs, Yellow – Leaderless CDSs, and Orange – All CDSs. **A.** Metagene plot of the SD affinity calculated using an 8 bp rRNA sequence 5′ CACCUCCU 3′ and the Free2bind software [16]. CDSs are centered at the start codon. 24.6% of start codons were preceded by a SD site, while the frequency of SD sites encoded in randomly generated sequences is 19.2%. **B.** Metagene plot of the average RNA folding energy calculated in a sliding 50 bp window with a step every 1 bp using the Vienna RNAfold package [72]. CDSs are centered at the start codon.
(TIF)

Figure S4 Codon dependent pausing in *C. crescentus*. **A.** Metagene plot of the average ribosome density across all highly expressed CDSs (>3 reads per codon) aligned relative to the start and stop codons. On average, there are peaks of ribosome occupancy at the start and stop codons. **B.** Average codon occupancy (at the ribosomal A-site) compared to the genomic codon abundance for each codon. The only codon with considerable pausing is AGU, read by tRNA^Ser^_GCU_. **C.** Pausing at AGU codons in abolished in PYE media. Rank of the fold change of pause intensity between M2G and PYE media. While three codons have a 2 fold change in codon occupancy, they were all low occupancy codons (<1 ribosome occupancy) and do not become strongly paused in PYE (<2 fold ribosome occupancy).
(TIF)

Figure S5Translation efficiency does not correlate with Shine-Dalgarno strength at start codon. Plot of the calculated SD strength vs the translation efficiency = 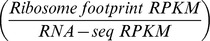 for genes expressed in M2G medium.(TIF)

Figure S6 Phase contrast images of *C. crescentus* cells overexpressing *ftsWs*. *C. crescentus* strain NA1000 harboring pBX*ftsWs*-SPA was grown in PYE media supplemented with kanamycin and induced with 0.3% xylose for 6 hours before imaging on PYE agarose pads. Black scale bar is 2 µM.
(TIF)

Figure S7 Distribution of operon sizes. Global distribution of the number of CDSs per operon.
(TIF)

Figure S8 Differential mRNA and translation levels in operons. Comparison of **A.** Ribosomes profiling and **B.** RNA-seq levels for the 2^nd^, 3^rd^, and 4^th^ CDSs in each operon with 4 or more CDSs compared to the level of the 1^st^ CDS. Data shown is for genes expressed in M2G.
(TIF)

Figure S9 Distribution of Shine-Dalgarno affinities for *B. subtilis* and *E. coli*. Calculated aSD affinity preceding the start codon for each CDS. Fraction of SD containing CDSs are 94.3% and 66.9% of CDSs in *B. subtilis* and *E. coli*., respectively [16].
(TIF)

Figure S10Equivalent translation of non-Shine-Dalgarno led and Shine-Dalgarno led mRNAs. Comparison of the translation efficiency = 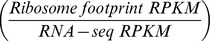 between leaderless, non-SD led, and SD led mRNAs. Data shown are for genes expressed in M2G.(TIF)

Figure S11 Verification of ribosome profiling derived coding DNA sequences using western blot. Cells were grown to mid log and induced with 0.3% xylose for 2 hours as indicated. Cells were harvested by centrifugation, lysed by boiling in 1× Laemmli sample buffer, and subjected to western blotting. Where applicable, bands of the expected size are highlighted with asterisks. **A.** **Verification of the start codon of** *ftsA* **.** Indicated cell lysates were blotted with anti-FtsA antibody. We used a deletion of the clpA protease (ΔclpA) which specifically degrades FtsA [79]. **B.** **Verification of** *ftsWs* **translation.** To verify the translation of the *ftsWs* isoform, we designed a plasmid (p*ftsWs*) which could only express the short form by replacing the upstream promoter with a strong transcription terminator. The CDS of *ftsWs* was then tagged with an in-frame C-terminal YFP and run on a western blot with anti-GFP antibody. The left lane shows that *ftsWs* is transcribed and translated from the internal promoter inside *ftsW*, yielding the FtsWs-YFP product and no appearance of the full length FtsW from this plasmid. As a control, XylX::YFP was induced with 0.3% xylose for 6 hours. **C.** **Verification of new small CDSs.** To verify the translation of the new small CDSs, we picked 4 small CDSs with different ribosome profiling densities and inserted an in-frame YFP into the chromosome and performed a western blot with anti-GFP antibody. For each of the 4 CDSs, we found bands that ran at sizes consistent with the predicted size.
(TIF)

Figure S12 Alignment of rRNA anti-Shine-Dalgarno sites. The sequences of the aSD are shown for *C. crescentus*, *B. subtilis*, and *E. coli*.
(TIF)

Figure S13 Occurrence of Shine-Dalgarno sites in CDSs. The normalized occurrence of each possible hexanucleotide sequence is plotted vs. the hexanucleotide affinity to the aSD. Stronger aSDs have lower occurrence in the CDSs, suggesting a negative selection against them.
(TIF)

Figure S14 Library preparation does not affect anti-Shine-Dalgarno pausing. RNA-seq read density is not enriched for peaks at SD sites as it is randomly sheared by base-hydrolysis. This suggests that the library preparation procedure does lead to the observed SD mediated ribosome pausing.
(TIF)

Figure S15 Protein levels correlate with ribosome profiling translation levels. To validate that the ribosome profiling read density measures the translation rate, we measured the relative protein levels of three genes with C-terminal YFP translational fusions. The band corresponding to the predicted molecular weight of each protein fusion is marked with an asterisk. We find that the relative band intensity correlates well with the ribosome profiling levels (R^2^ = 0.96).
(TIF)

Figure S16 Morphology of pBX-*ftsAΔN1-18* cells after extended overexpression. Cells were grown in PYE kanamycin with 0.3% xylose and imaged with phase-contrast microscopy at the indicated times. Black scale bar is 6.05 µm.
(TIF)

Table S1 Expressed riboswitch elements. Putative riboswitches were determined by searching 5′ UTRs in the Rfam database [35].
(DOCX)

Dataset S1 New annotations of the *C. crescentus* genome. Compiled CDS, ncRNA, and operon maps are found here. Other features, such as leaderless mRNAs and putative alternative start codons, are also deposited here. Additionally, translation and RNA levels in each media are deposited here.
(XLSX)

Dataset S2 Pooled ribosome profiling data used for genome mapping – forward strand data. Ribosome footprints from M2G and PYE were pooled to create a comprehensive dataset for mapping CDSs. This file contains the forward strand data. First column contains the nucleotide position in the genome, and the second column contains the center weighted ribosome footprint count.
(RAR)

Dataset S3 Pooled ribosome profiling data used for genome mapping – reverse strand data. Ribosome footprints from M2G and PYE were pooled to create a comprehensive dataset for mapping CDSs. This file contains the reverse strand data. First column contains the nucleotide position in the genome, and the second column contains the center weighted ribosome footprint count.
(RAR)

Dataset S4 Pooled RNA-seq data used for genome mapping – forward strand data. RNA-seq reads from M2G and PYE were pooled to create a comprehensive dataset for mapping RNAs. This file contains the forward strand data. First column contains the nucleotide position in the genome, and the second column contains the number of reads mapped to the 5′ nucleotide.
(RAR)

Dataset S5 Pooled RNA-seq data used for genome mapping – reverse strand data. RNA-seq reads from M2G and PYE were pooled to create a comprehensive dataset for mapping RNAs. This file contains the reverse strand data. First column contains the nucleotide position in the genome, and the second column contains the number of reads mapped to the 5′ nucleotide.
(RAR)

Dataset S6 Systematic analysis of *E. coli* and *B. subtilis* CDSs. Ribosome profiling data from *E. coli* MG1655 and *B. subtilis* 168 [10] were analyzed using our CDS searching scheme (Materials and Methods).
(XLSX)

Dataset S7 Comparison of RNA-seq transcripts with northern blots. RNA-seq derived transcripts were compared to northern blots from [4] to validate our RNA-seq mapping approach.
(XLSX)
