# Supplementary figures and images for: The Coding and Noncoding Architecture of the Caulobacter crescentus Genome
Source: PLoS Genet. 2014 Jul 31;10(7):e1004463. doi: 10.1371/journal.pgen.1004463 (PMC4117421; doi:10.1371/journal.pgen.1004463)

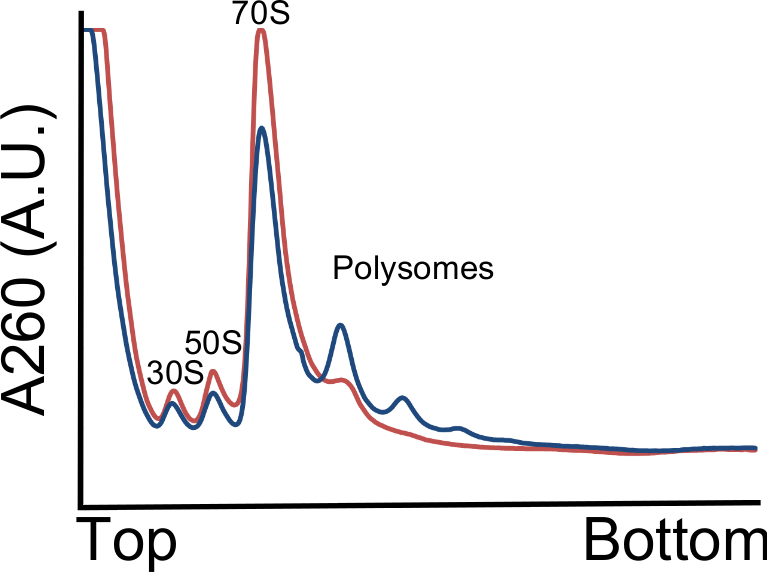

Supplement: Figure S1 — C. crescentus polysome digestion with micrococcal nuclease. Absorbance at 260 nm of polyribosomes separated on a 10 to 55% sucrose gradient before (blue) or after (red) digestion with micrococcal nuclease. After digestion the 70S peak was purified and the resulting mRNA footprints were prepared for high-throughput sequencing. (TIF) [file pgen.1004463.s001.tif]

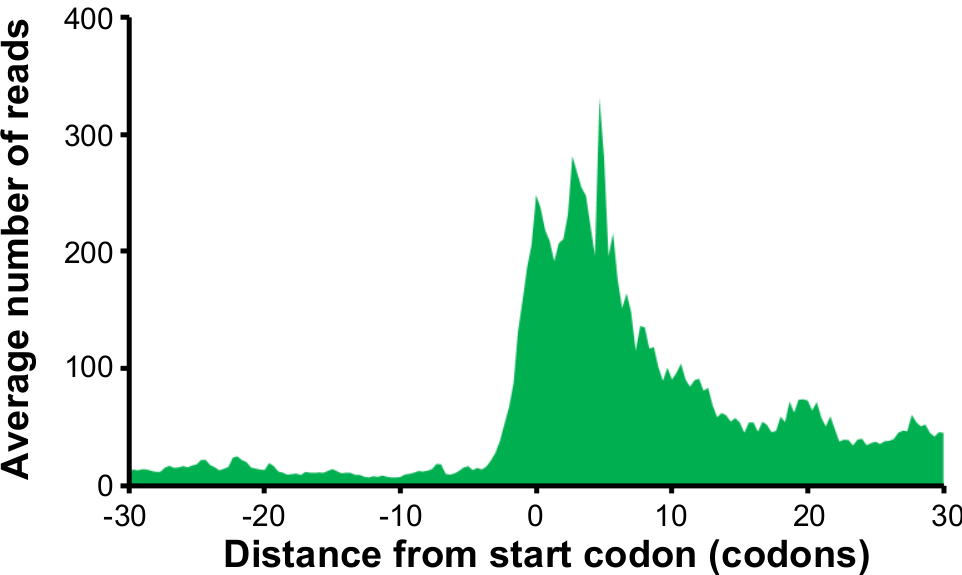

Supplement: Figure S2 — Metagene plot of ribosome density at start codons. The average ribosome density plotted at CDSs where start codons were verified by LC-MS. (TIF) [file pgen.1004463.s002.tif]

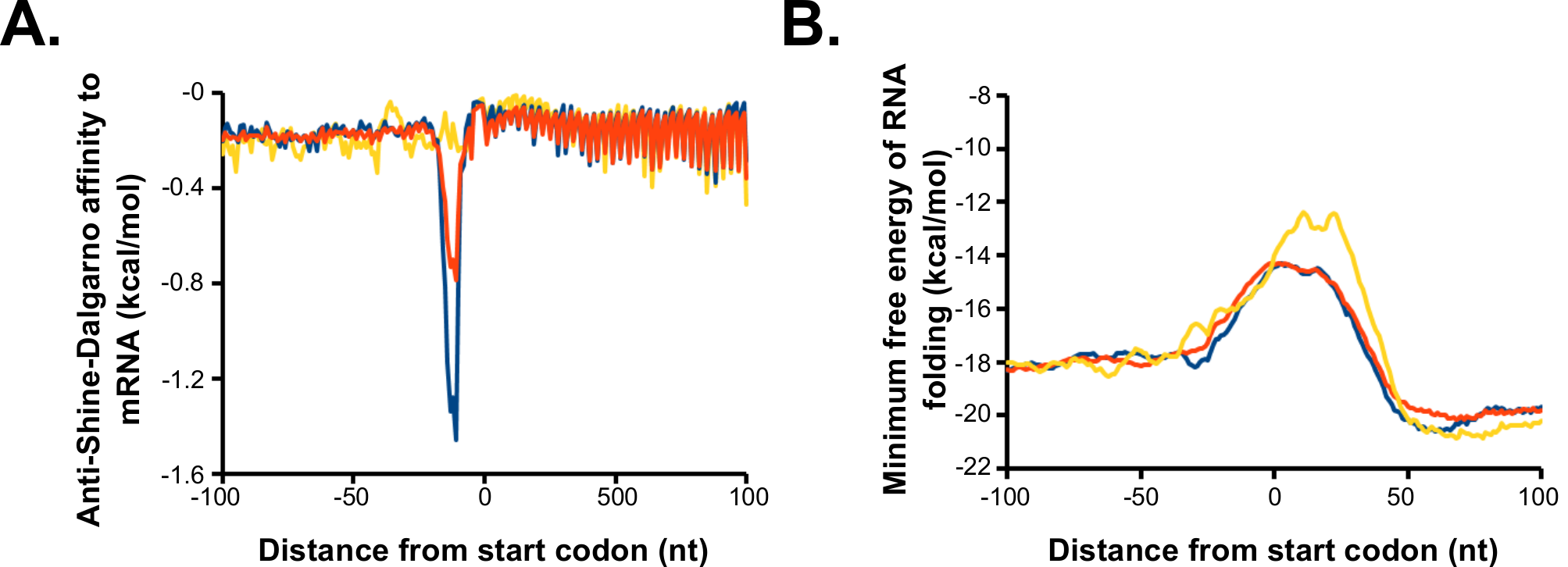

Supplement: Figure S3 — RNA folding energy and Shine-Dalgarno affinity to rRNA for C. crescentus translation initiation sites. Blue – SD containing CDSs, Yellow – Leaderless CDSs, and Orange – All CDSs. A. Metagene plot of the SD affinity calculated using an 8 bp rRNA sequence 5′ CACCUCCU 3′ and the Free2bind software [16]. CDSs are centered at the start codon. 24.6% of start codons were preceded by a SD site, while the frequency of SD sites encoded in randomly generated sequences is 19.2%. B. Metagene plot of the average RNA folding energy calculated in a sliding 50 bp window with a step every 1 bp using the Vienna RNAfold package [72]. CDSs are centered at the start codon. (TIF) [file pgen.1004463.s003.tif]

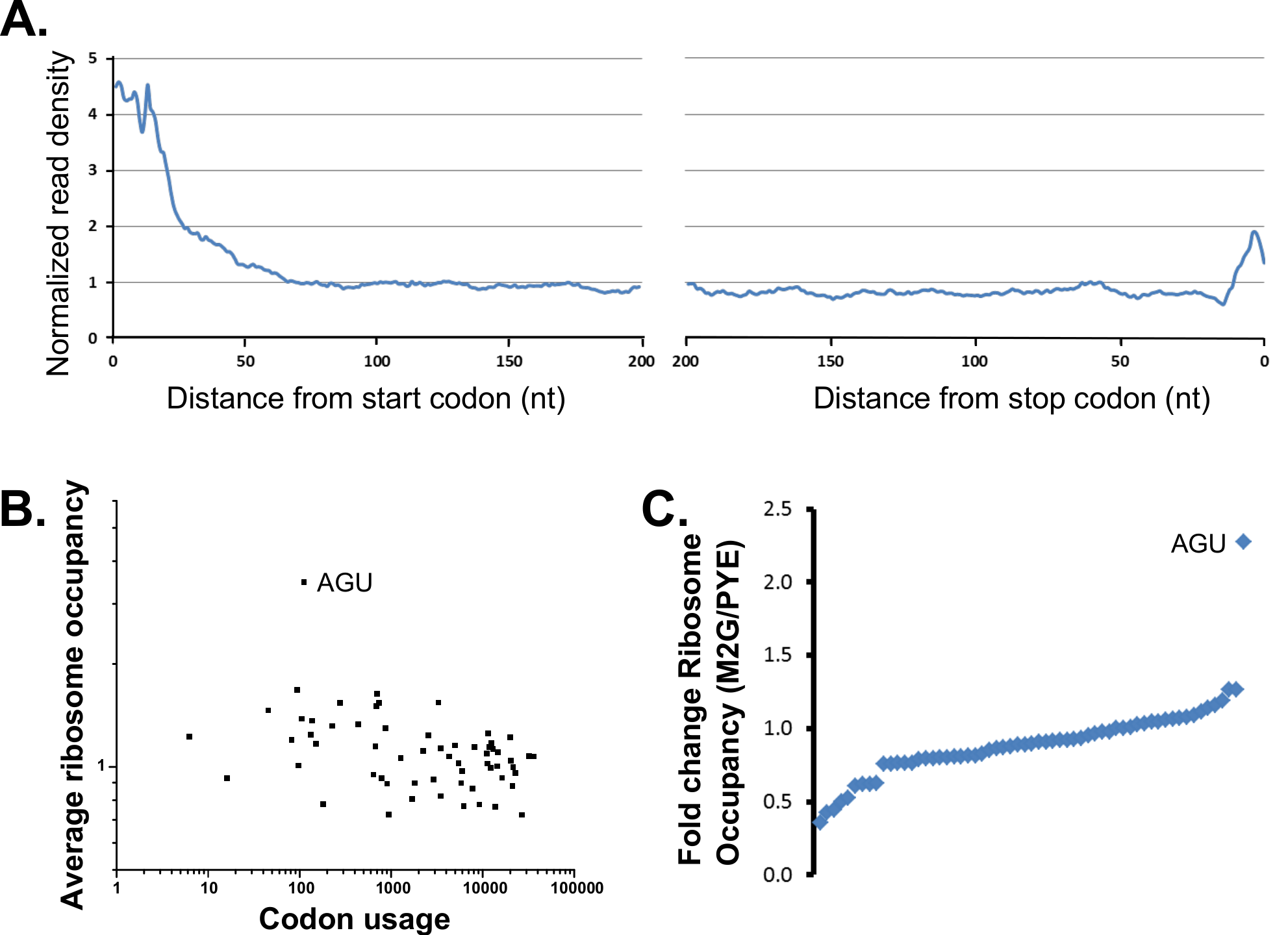

Supplement: Figure S4 — Codon dependent pausing in C. crescentus. A. Metagene plot of the average ribosome density across all highly expressed CDSs (>3 reads per codon) aligned relative to the start and stop codons. On average, there are peaks of ribosome occupancy at the start and stop codons. B. Average codon occupancy (at the ribosomal A-site) compared to the genomic codon abundance for each codon. The only codon with considerable pausing is AGU, read by tRNASer GCU. C. Pausing at AGU codons in abolished in PYE media. Rank of the fold change of pause intensity between M2G and PYE media. While three codons have a 2 fold change in codon occupancy, they were all low occupancy codons (<1 ribosome occupancy) and do not become strongly paused in PYE (<2 fold ribosome occupancy). (TIF) [file pgen.1004463.s004.tif]

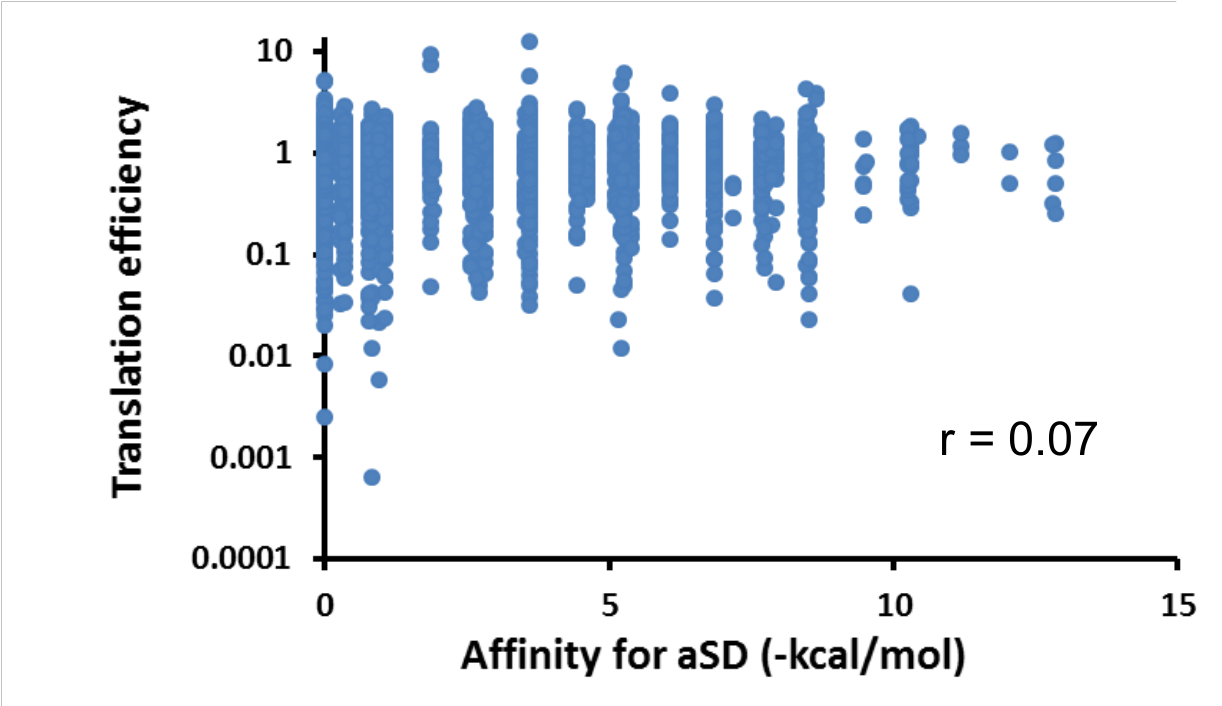

Supplement: Figure S5 — Translation efficiency does not correlate with Shine-Dalgarno strength at start codon. Plot of the calculated SD strength vs the translation efficiency = for genes expressed in M2G medium. (TIF) [file pgen.1004463.s005.tif]

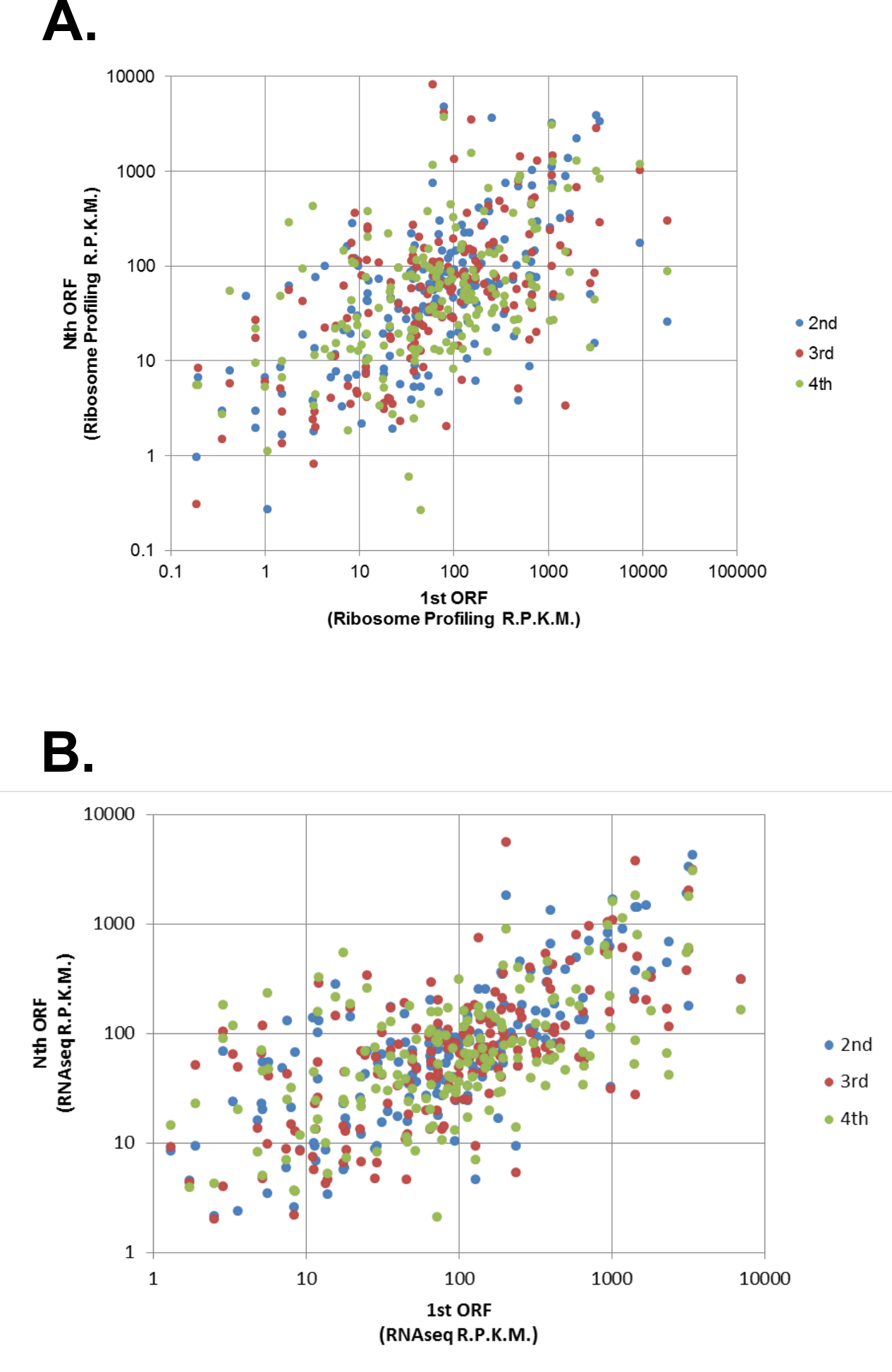

Supplement: Figure S8 — Differential mRNA and translation levels in operons. Comparison of A. Ribosomes profiling and B. RNA-seq levels for the 2nd, 3rd, and 4th CDSs in each operon with 4 or more CDSs compared to the level of the 1st CDS. Data shown is for genes expressed in M2G. (TIF) [file pgen.1004463.s008.tif]

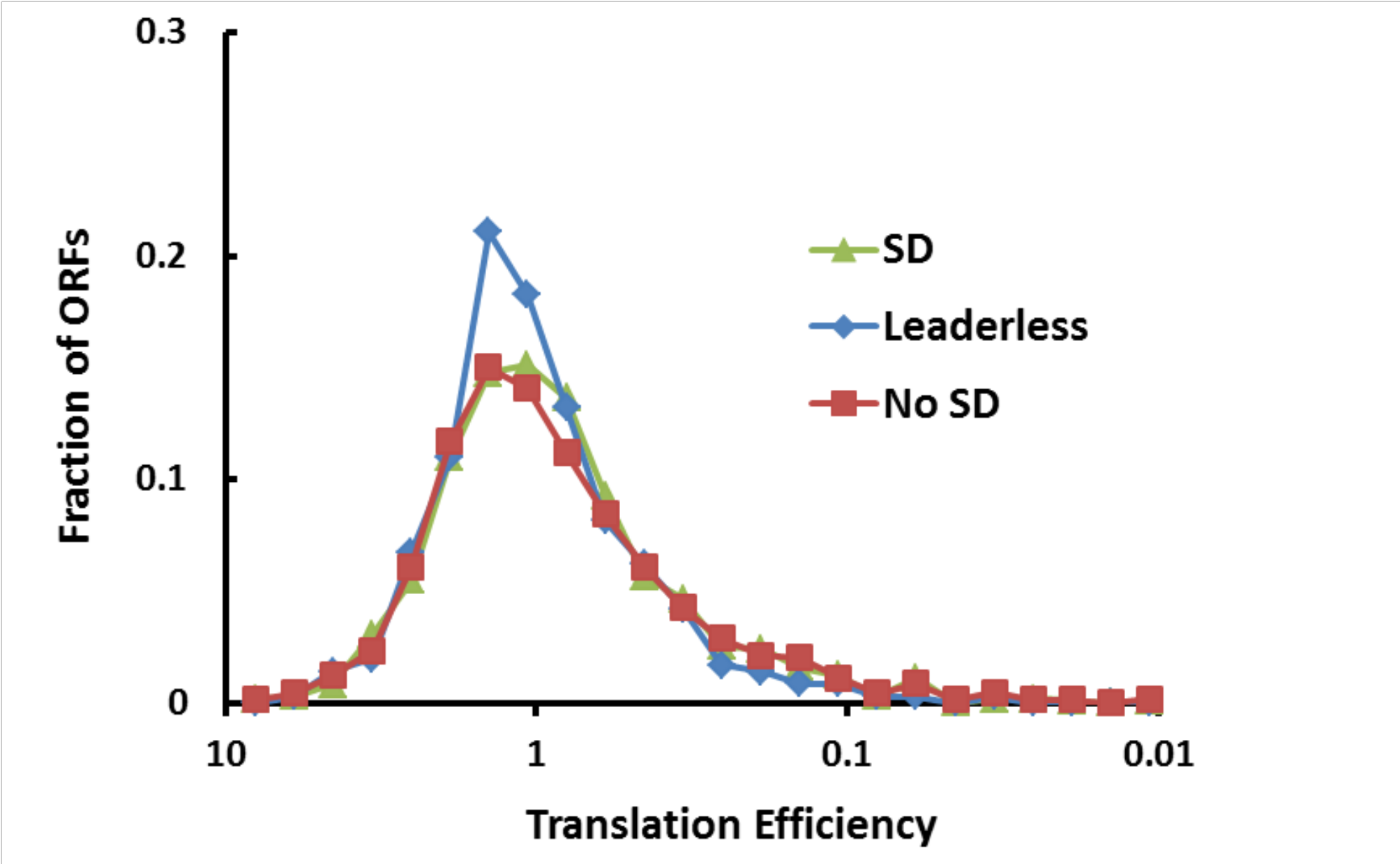

Supplement: Figure S9 — Distribution of Shine-Dalgarno affinities for B. subtilis and E. coli. Calculated aSD affinity preceding the start codon for each CDS. Fraction of SD containing CDSs are 94.3% and 66.9% of CDSs in B. subtilis and E. coli., respectively [16]. (TIF) [file pgen.1004463.s009.tif]

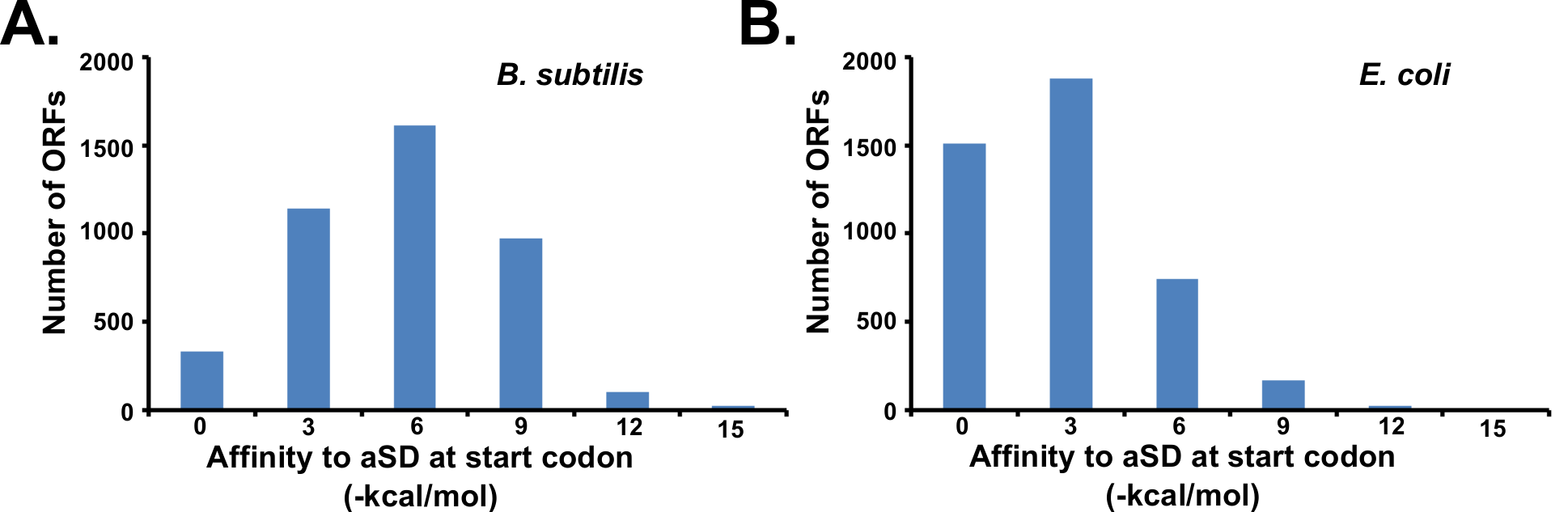

Supplement: Figure S10 — Equivalent translation of non-Shine-Dalgarno led and Shine-Dalgarno led mRNAs. Comparison of the translation efficiency = between leaderless, non-SD led, and SD led mRNAs. Data shown are for genes expressed in M2G. (TIF) [file pgen.1004463.s010.tif]

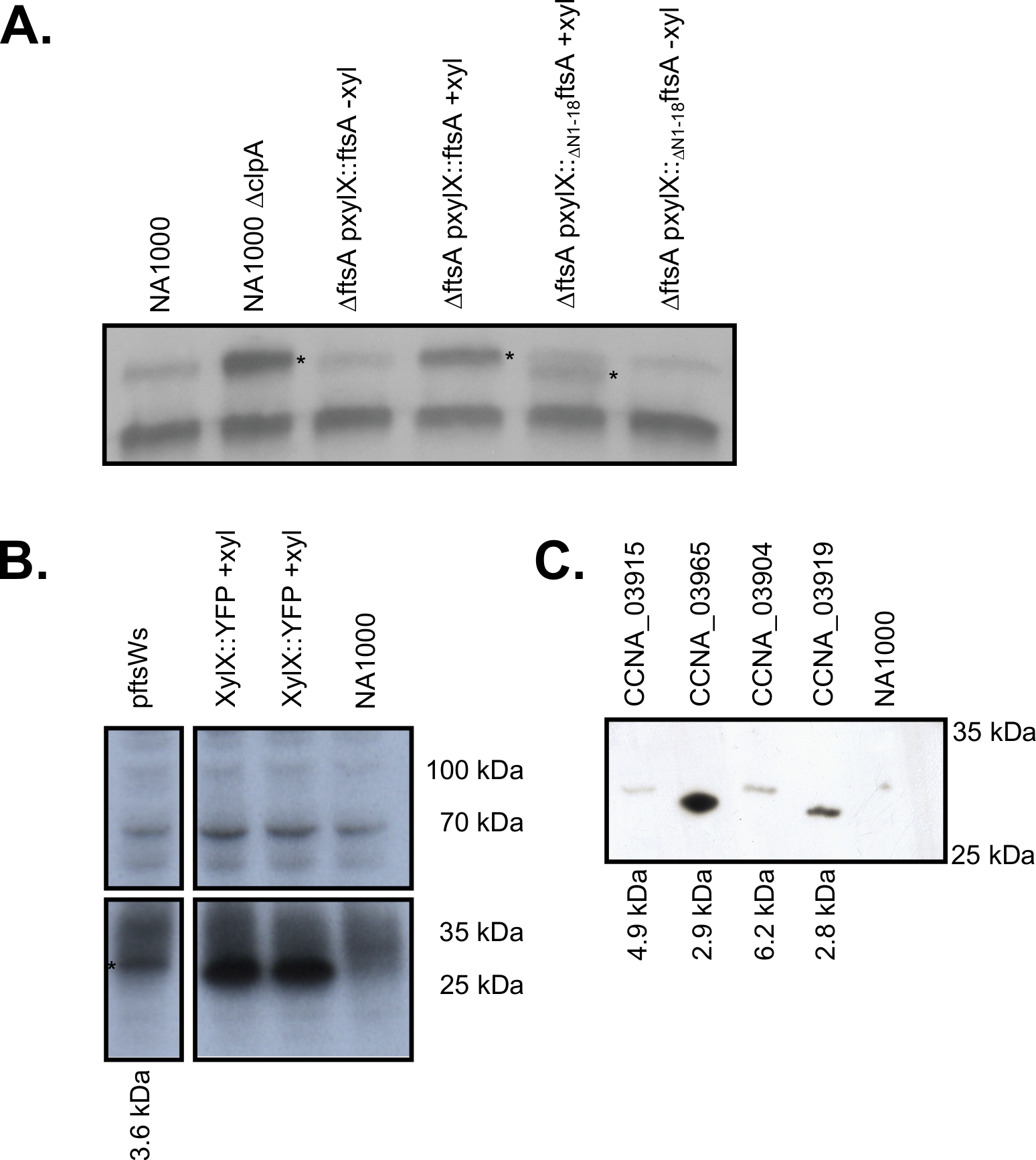

Supplement: Figure S11 — Verification of ribosome profiling derived coding DNA sequences using western blot. Cells were grown to mid log and induced with 0.3% xylose for 2 hours as indicated. Cells were harvested by centrifugation, lysed by boiling in 1× Laemmli sample buffer, and subjected to western blotting. Where applicable, bands of the expected size are highlighted with asterisks. A. Verification of the start codon of ftsA . Indicated cell lysates were blotted with anti-FtsA antibody. We used a deletion of the clpA protease (ΔclpA) which specifically degrades FtsA [79]. B. Verification of ftsWs translation. To verify the translation of the ftsWs isoform, we designed a plasmid (pftsWs) which could only express the short form by replacing the upstream promoter with a strong transcription terminator. The CDS of ftsWs was then tagged with an in-frame C-terminal YFP and run on a western blot with anti-GFP antibody. The left lane shows that ftsWs is transcribed and translated from the internal promoter inside ftsW, yielding the FtsWs-YFP product and no appearance of the full length FtsW from this plasmid. As a control, XylX::YFP was induced with 0.3% xylose for 6 hours. C. Verification of new small CDSs. To verify the translation of the new small CDSs, we picked 4 small CDSs with different ribosome profiling densities and inserted an in-frame YFP into the chromosome and performed a western blot with anti-GFP antibody. For each of the 4 CDSs, we found bands that ran at sizes consistent with the predicted size. (TIF) [file pgen.1004463.s011.tif]

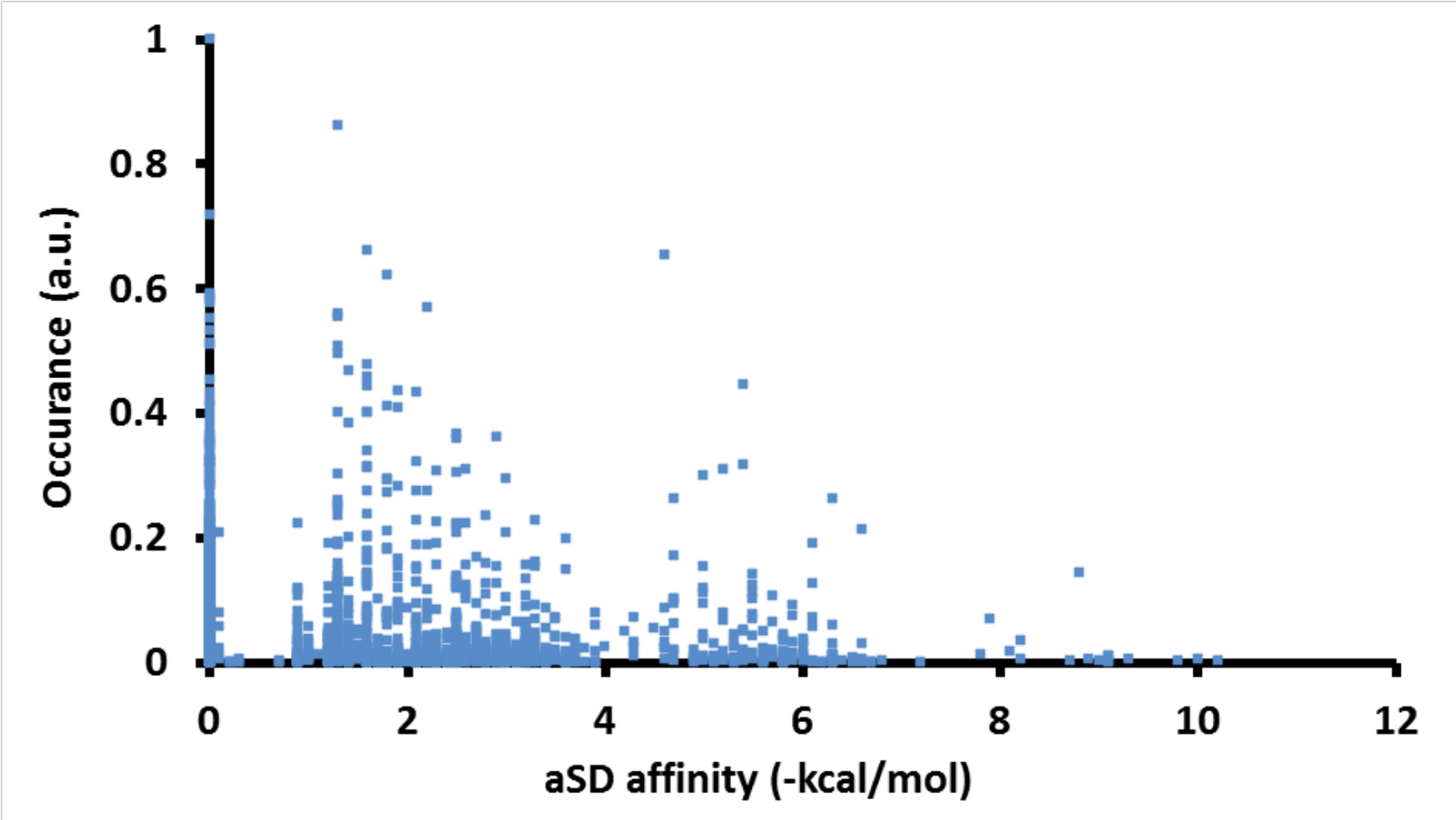

Supplement: Figure S13 — Occurrence of Shine-Dalgarno sites in CDSs. The normalized occurrence of each possible hexanucleotide sequence is plotted vs. the hexanucleotide affinity to the aSD. Stronger aSDs have lower occurrence in the CDSs, suggesting a negative selection against them. (TIF) [file pgen.1004463.s013.tif]

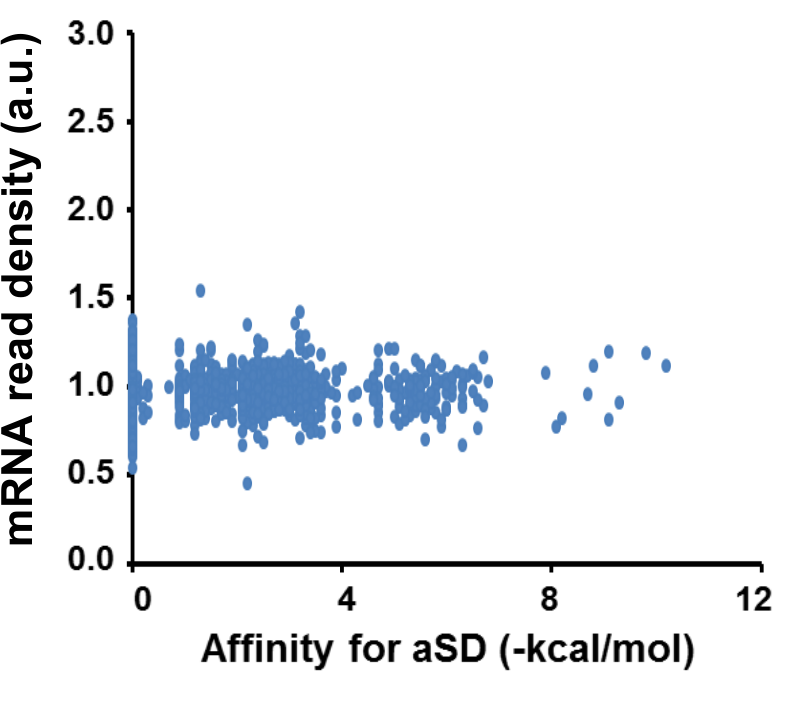

Supplement: Figure S14 — Library preparation does not affect anti-Shine-Dalgarno pausing. RNA-seq read density is not enriched for peaks at SD sites as it is randomly sheared by base-hydrolysis. This suggests that the library preparation procedure does lead to the observed SD mediated ribosome pausing. (TIF) [file pgen.1004463.s014.tif]

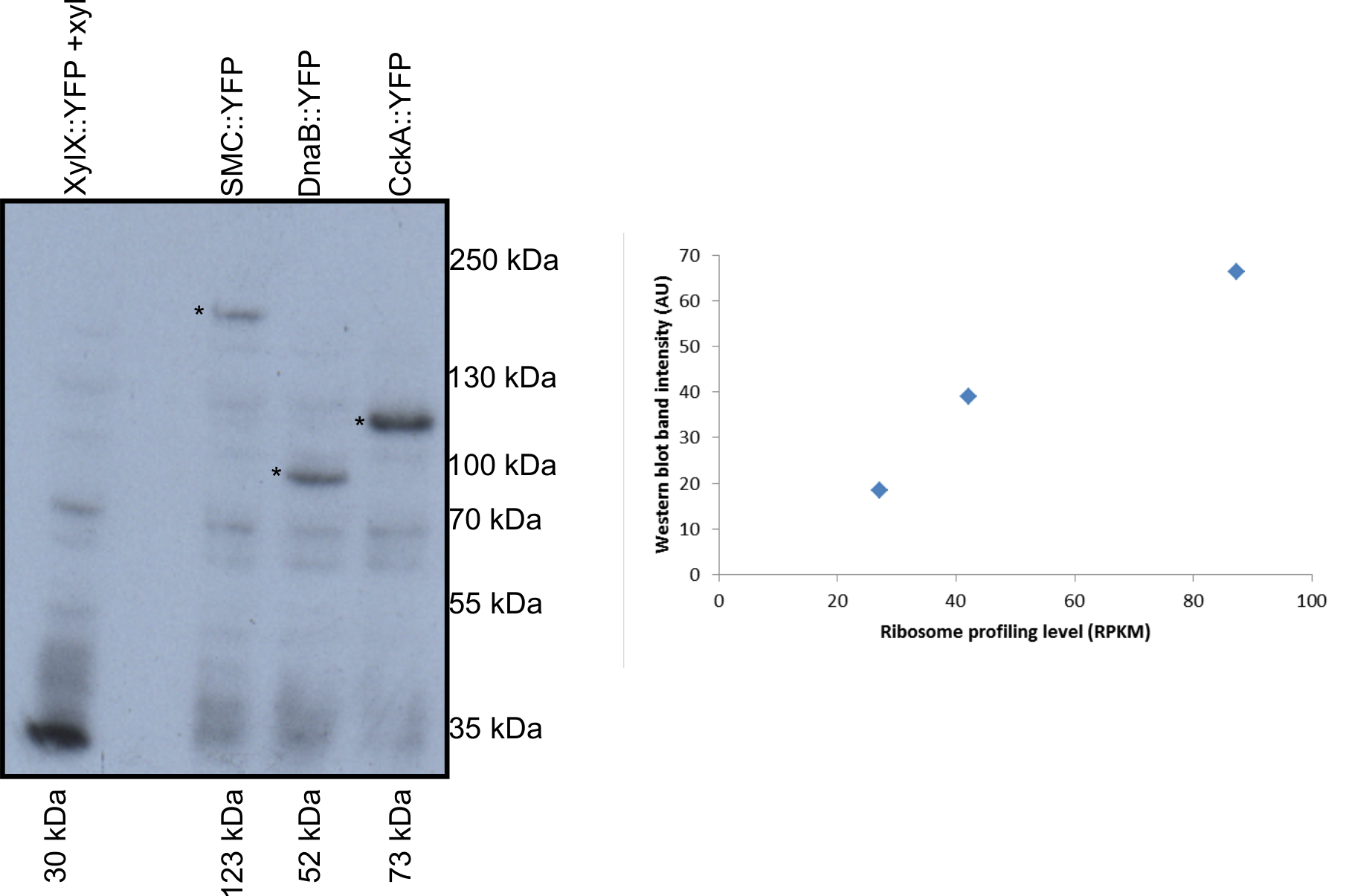

Supplement: Figure S15 — Protein levels correlate with ribosome profiling translation levels. To validate that the ribosome profiling read density measures the translation rate, we measured the relative protein levels of three genes with C-terminal YFP translational fusions. The band corresponding to the predicted molecular weight of each protein fusion is marked with an asterisk. We find that the relative band intensity correlates well with the ribosome profiling levels (R2 = 0.96). (TIF) [file pgen.1004463.s015.tif]
