# Supplementary material for: The Coding and Noncoding Architecture of the Caulobacter crescentus Genome
Source: PLoS Genet. 2014 Jul 31;10(7):e1004463. doi: 10.1371/journal.pgen.1004463 (PMC4117421; doi:10.1371/journal.pgen.1004463)
Supplement: Table S1 — Expressed riboswitch elements. Putative riboswitches were determined by searching 5′ UTRs in the Rfam database [35]. (DOCX) [file pgen.1004463.s017.docx]

| Supplemental Table 1. **Expressed conserved riboswitch elements.** | | | |
| --- | --- | --- | --- |
| **Gene** | **Name** | **Riboswitch** | **Rfam ID** |
| CCNA_03465 | aminomethyltransferase | Glycine | RF00504 |
| CCNA_01826 | Vitamin B12 receptor | Cobalamin | RF00174 |
| CCNA_00515 | *metE* | Cobalamin | RF00174 |
| CCNA_02109 | *thiC* | Thiamin pyrophosphate (TPP) | RF00059 |
